# Effects of time delay on excited quarter- and half-car models with jumping nonlinearities

**DOI:** 10.1371/journal.pone.0340370

**Published:** 2026-02-05

**Authors:** Masahisa Watanabe, Manish D. Shrimali, Awadhesh Prasad

**Affiliations:** 1 Division of Environmental and Agricultural Engineering, Institute of Agriculture, Tokyo University of Agriculture and Technology, Tokyo, Japan; 2 Department of Physics and Astrophysics, University of Delhi, New Delhi, India; 3 Department of Physics, Central University of Rajasthan, Ajmer, India; National University of Singapore, SINGAPORE

## Abstract

Nonlinear vehicle dynamics are omnipresent and significantly affect driving performance and safety. Jumping vehicles, in particular, exhibit strong nonlinearities that can lead to severe vibrations and steering instabilities. This study investigates the dynamics of nonlinear jumping vehicle models with and without time delay to clarify their fundamental characteristics. Quarter- and half-car models with jumping nonlinearity are considered, and delayed feedback is introduced as an active suspension control. Numerical simulations under periodic and random excitations reveal several key findings as follows. Both models exhibit sudden, discontinuous transitions from periodic to chaotic dynamics in their bifurcation diagrams. A general relationship between the time period of periodic motions and the forcing period is also identified and further validated using a forced Duffing oscillator with time delay, confirming its generic nature. With the introduction of delay, both in-phase and out-of-phase motions emerge between the front and rear, even under simultaneous excitation. From a practical standpoint, in-phase motion is undesirable, making the realization of out-of-phase behavior within a specific delay range important for improving ride performance. This study identifies the stability regions of nonlinear quarter- and half-car models using bifurcation diagrams, Lyapunov exponents, and frequency response curves. In addition, the inclusion of a time delay is shown to effectively stabilize chaotic motions into periodic responses and to induce both in-phase and out-of-phase oscillations. These findings demonstrate that time delay plays a significant role in enhancing the stability of vehicle models with jumping nonlinearities.

## 1 Introduction

The theory of nonlinear dynamics and chaos has been applied in engineering fields, including cutting machines [1,2], bipedal robots [3,4], aircraft [5,6], piezoelectric devices [7,8], and space satellites [9,10]. Chaos, characterized by complex and unpredictable behavior, is generally undesirable in many engineering systems. Consequently, numerous chaos control techniques, such as OGY control [11], time-delayed feedback [12], sliding mode control [13], and linear augmentation [14], have been proposed to suppress chaotic dynamics. Ground vehicle dynamics is another important area where nonlinear dynamics and chaos theory have been actively applied [15]. Nonlinear vehicle models are known to exhibit complex responses, including chaotic and quasi-periodic motions. Since chaotic motion can lead to unstable behavior and potentially hazardous situations, various studies have applied chaos control methods to vehicle systems to convert chaotic oscillations into predictable periodic responses [16].

Various vehicle models, such as vehicle steering systems [17], ride vibrations [15], and traction models [18] have been investigated in terms of nonlinear dynamics and chaos. These models correspond to the lateral, vertical, and longitudinal motions of the vehicles. The steering system model describes the lateral motion of a vehicle on a plane. The simplest description of the steering system is the bicycle model, which assumes that the roll motion is negligible and that the slip angle is the same for the left and right tires [19]. The four-wheel steering model does not assume that the right and left wheels are identical [20]. Several investigations have been carried out into nonlinear steering system models. Bifurcation and stability analyses were performed on a bicycle model in a plane [17]. Hopf bifurcation was identified in a nonlinear four-wheel steering system using a driver model [21]. Chaotic and quasi-periodic oscillations were observed in a nonlinear vehicle steering system model of a cubic cornering force with driver control [22]. The stability of the nonlinear vehicle model in the plane was analyzed using the Lyapunov exponent concept [23]. The chaotic behavior in the steering model of an electric vehicle was stabilized by adaptive time-delayed feedback control in Ref. [24]. The ride vibration model describes the vertical vibrations of vehicles and passengers [25]. Nonlinear ride vibration models have been investigated for various types of vehicles, such as passenger vehicles [15] and agricultural tractors [26]. The simplest description of a nonlinear ride vibration model is the quarter-car model, which considers only the vertical, or heave, motions. The half-car model had front and rear wheels, considering the heave and pitch motions. The full-car model had left and right wheels as well as front and rear wheels, considering the heave, pitch, and roll motions. The quarter-car model with cubic suspension stiffness and damping was investigated under periodic and stochastic road excitations [27,28]. Vehicle tires may occasionally lose contact with the supporting surface, leading to a phenomenon referred to by the authors as “jumping nonlinearity [29].” In our previous study, the authors investigated the impact dynamics of a quarter-car model exhibiting this jumping nonlinearity and identified the occurrence of chaotic and quasi-periodic vibrations. Chaotic vibrations and bifurcations were observed in half- and full-car models with exponential stiffness and piecewise linear damping [15,30]. Chaotic motions were also observed in a full-vehicle model using a magnetorheological damper [31]. In investigations on nonlinear vehicle dynamics, chaos control is achieved through active or semi-active suspension control, which is a popular technology for reducing ride vibrations in commercial cars. In practice, active suspension systems are physically implemented using external energy sources, most commonly hydraulic actuators [32–34], which provide the necessary force to actively control vehicle dynamics. Typical applications of chaos control in vehicle systems include pulsive control [16], delayed feedback [29,35], and fuzzy terminal sliding-mode control [36]. The traction model describes the longitudinal motion of a vehicle during acceleration or braking [37]. Compared with steering and ride vibration models, few investigations have been conducted on nonlinear traction or longitudinal dynamics models. Braking and acceleration in a nonlinear single-wheel traction model were investigated, and a bifurcation analysis was performed to identify vehicle stability [18]. Longitudinal self-excited vibration, which is known as a power hop, was modeled using a stick-slip in Ref. [38].

Among nonlinearities in vehicle dynamics, jumping nonlinearity is significantly dangerous for driving because tires lose contact with the ground during jumping, resulting in steering instability and uncontrollability [39–41]. In particular, chaotic vibrations can induce a large, unpredictable jump and severe ride vibrations owing to the impact between the tire and the ground. Despite their importance in driving safety and performance, jumping vehicle dynamics have not been studied as much as other nonlinear ride vibration models. Chaotic vibrations should be stabilized using chaos control techniques to enhance the stability of jumping vehicles. In general, time-delayed interactions play a crucial role in controlling complex dynamics in nonlinear systems. Therefore, extensive research has been conducted on time-delayed nonlinear systems, such as conjugate coupling [42], coupled oscillators [43], q-deformed logistic maps [44], and coupled discrete maps [45]. In addition to these theoretical investigations, time-delayed nonlinear systems have also been studied in various practical models, including genetic regulatory systems [46], electrical circuits [47], food chain systems [48], and neural systems [49]. In a previous study, we also applied delayed feedback to a quarter-car model with jumping nonlinearity through an active suspension control and eliminated chaotic vibrations [29,35]. However, the study of jumping vehicle dynamics and its control is in the early stages and should be investigated further to enhance vehicle performance and stability. In particular, it is necessary to understand the effects of delays on higher order jumping vehicle models. The primary objective of the present study is to understand the general characteristics of nonlinear jumping vehicle models with a delay in improving the driving stability of vehicles. The half-car model with jumping nonlinearity is discussed, as is the quarter-car model in this study, in which sudden discontinuous transitions in the bifurcation diagrams are observed. A general relationship between the period of periodic motion and the forcing period is observed. The in-phase and out-of-phase motions in the systems are also observed with and without delay. The remainder of this paper is organized as follows: In Section 2, the nonlinear jumping vehicle models are discussed. In Section 3, the numerical simulations performed for quarter- and half-car models with and without delay under periodic excitations are presented. The primary findings are summarized in Section 4.

## 2 Nonlinear jumping vehicle models

### 2.1 Quarter-car model

**Fig 1:**
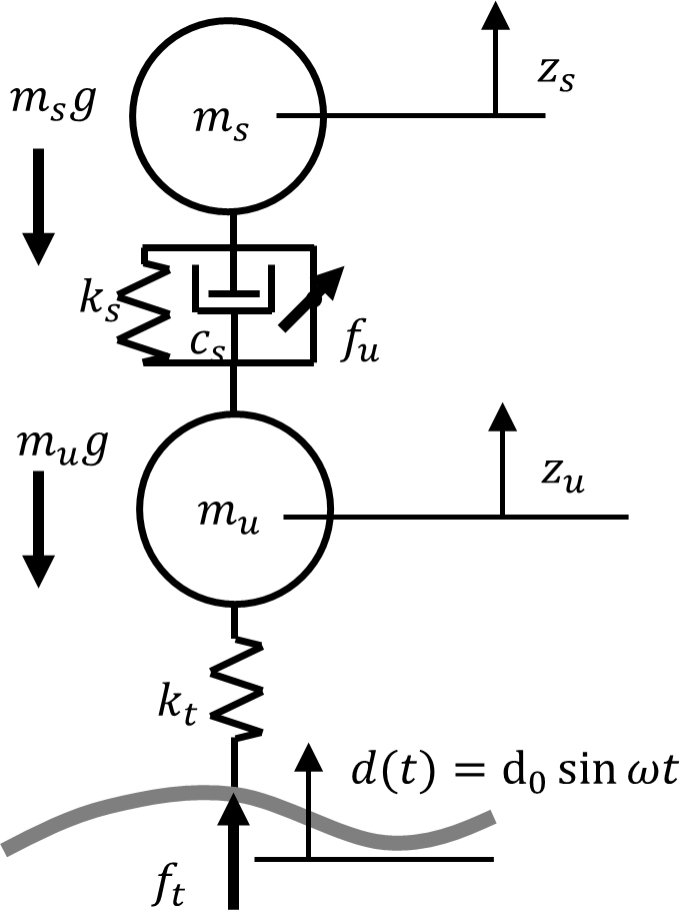
Schematic illustration of the quarter-car model (See Appendix A.1 for symbols and detaielsS1 File).

This section presents the quarter- and half-car models with jumping nonlinearity. The quarter-car model provides the simplest representation of vehicle dynamics and is commonly used to analyze vertical (heave) motion. The quarter-car model is a one-wheel model with two degrees of freedom, that is, the motions of the sprung mass z_s_ and unsprung mass z_u_. A schematic illustration of the quarter-car model is shown in Fig 1.

The equations of motion for the quarter-car model are as follows:


$$\begin{array}{c} m_{s}z_{s}^{{}^{\prime \prime }}=f_{s}+f_{u}-m_{s}g,\;\\ m_{u}z_{u}^{{}^{\prime \prime }}=f_{t}-f_{s}-f_{u}-m_{u}g, \end{array}$$ (1)

where the prime denotes differentiation with respect to time *t*, and *f*_s_, *f*_t_, and *f*_u_ are the suspension, tire, and control forces generated by an active suspension, respectively. The dimensionless forms (see Appendix A.1 in S1 File) of the equations of motion are as follows:


$$\begin{array}{c} {\ddot{Z}}_{s}=-{(\text{1}+\alpha )Z}_{s}-\text{2}\zeta (\text{1}+\alpha ){\dot{Z}}_{s}+{\alpha \beta Z}_{u}+(\text{1}+\alpha )u,\\ \;{\ddot{Z}}_{u}=\alpha Z_{s}+\text{2}\zeta \alpha {\dot{Z}}_{s}-{\alpha \beta Z}_{u}-\gamma +d_{\text{0}}\Omega^{\text{2}}\text{sin}\Omega t_{s}-\alpha u, \end{array}$$ (2)

where the dot denotes differentiation with respect to dimensionless time t_qs,_ which is defined as follows:


$$\begin{array}{c} t_{qs}=\omega_{qs}t,\;\text{and}\\ \omega_{qs}=\sqrt{\frac{k_{s}}{m_{s}}}. \end{array}$$ (3)

Dimensionless parameter β is introduced as follows to describe the jumping nonlinearity of the vehicle:



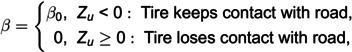

(4)


where β_0_ denotes the value of the stiffness ratio when the tire maintains contact with the ground, that is, when Z_u_ is negative. Otherwise, β is zero when the tire loses contact with the ground.

An active suspension is generally used to introduce a control force into a vehicle system. This feedback control can be a delay type [50]. The delay term can be easily implemented in experimental systems [51]. In this study, we consider the delayed control input to be as follows:


$$u=\varepsilon \left(Z_{u}(t)-Z_{u}(t-\tau )\right),$$ (5)

where ε and τ denote the feedback gain and delay time, respectively. Here, u is a dimensionless control input force. Please see Appendix A.1 in S1 File for the detailed derivation. The motion of the unsprung mass, Z_u_, is used for delayed feedback. This is a major source of nonlinearity; hence, the effect of the delay could be interesting.

### 2.2 Half-car model

**Fig 2:**
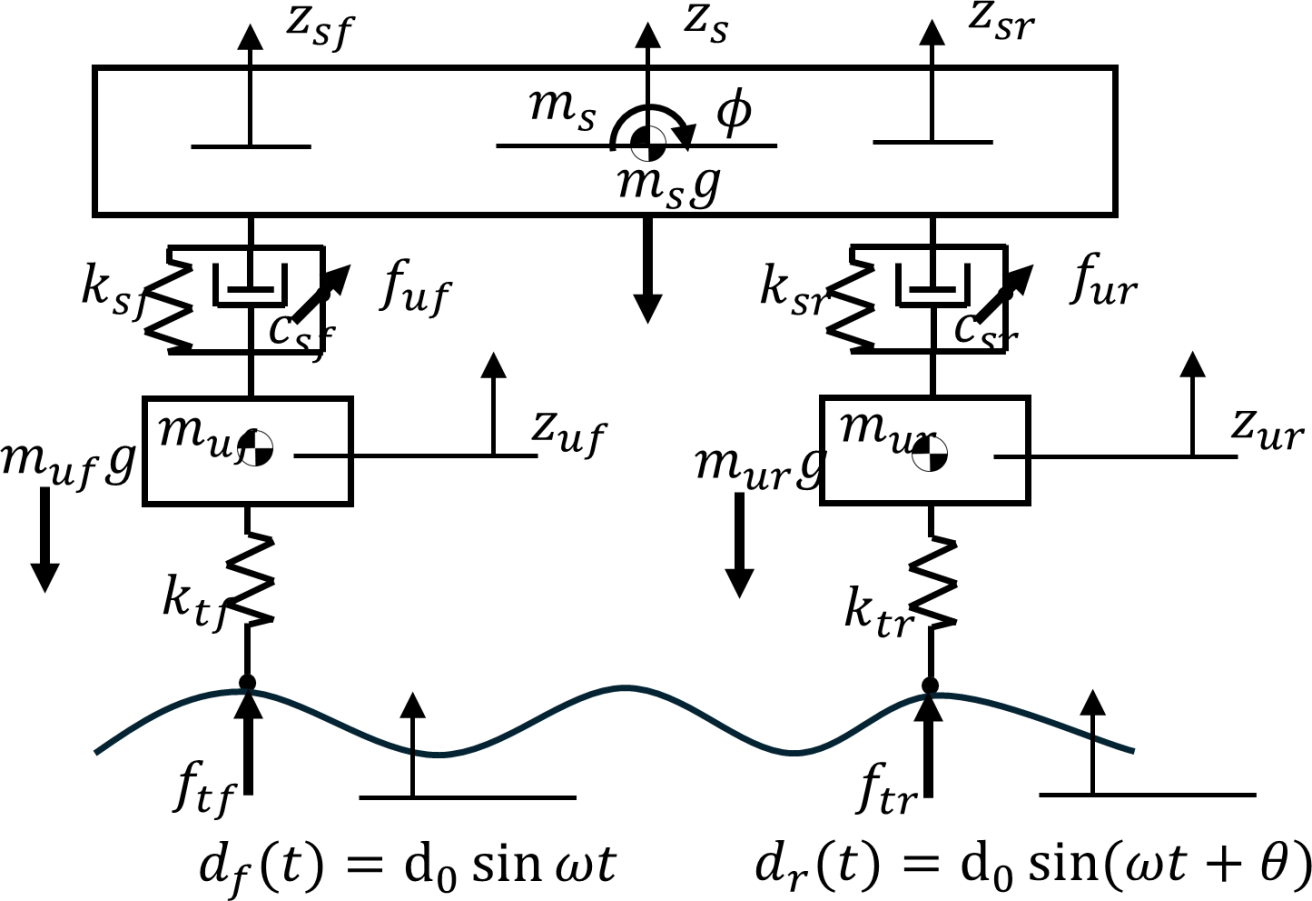
Schematic illustration of half-car model (See Appendix A.2 inS1 File for symbols and details).


In this section, a half-car model with jumping nonlinearity is presented. Unlike the quarter-car model, which captures only vertical (heave) motion, the half-car model incorporates both heave and pitch motions. The half-car model is a two-wheel model that has four degrees of freedom, sprung mass z_s_, pitch motion θ, front unsprung mass z_uf_, and rear unsprung mass z_ur_. The front sprung mass motion z_sf_ and rear sprung mass z_sr_ are represented by $z_{sf}=z+l_{f}\phi$ and $z_{sr}=z-l_{r}\phi$, respectively. The half-car model can be considered as a quarter-car model coupled between the front and rear positions. A schematic illustration of the half-car model is shown in Fig 2. The dimensionless equations of motion for the model are as follows (see Appendix A.2 in S1 File):

$$\begin{array}{cc} & {\ddot{Z}}_{sf}=-\beta_{sf}A_{f}Z_{sf}-\text{2}\zeta_{sf}A_{f}{\dot{Z}}_{sf}-\beta_{sr}A_{fr}Z_{sr}-\text{2}\zeta_{sr}A_{fr}{\dot{Z}}_{sr}+{\alpha_{f}\beta }_{tf}Z_{uf}+{A_{f}u}_{f}+A_{fr}u_{r},\\ & {\ddot{Z}}_{sr}=-\beta_{sf}A_{fr}Z_{sf}-\text{2}\zeta_{sf}A_{fr}{\dot{Z}}_{sf}-\beta_{sr}A_{r}Z_{sr}-\text{2}\zeta_{sr}A_{r}{\dot{Z}}_{sr}+{\alpha_{r}\beta }_{tr}Z_{ur}+{A_{fr}u}_{f}+A_{r}u_{r},\;\\ & {\ddot{Z}}_{uf}={\alpha_{f}\beta }_{sf}Z_{sf}+\text{2}\alpha_{f}\zeta_{sf}{\dot{Z}}_{sf}-{\alpha_{f}\beta }_{tf}Z_{uf}-\gamma_{h}+D_{\text{0}}\Omega^{\text{2}}\text{sin}\left(\Omega t_{\text{1}}\right)-{\alpha_{f}u}_{f},\;\\ & {\ddot{Z}}_{ur}={\alpha_{r}\beta }_{sr}Z_{sr}+\text{2}\alpha_{r}\zeta_{sr}Z_{sr}-{\alpha_{r}\beta }_{tr}Z_{ur}-\gamma_{h}+D_{\text{0}}\Omega^{\text{2}}\text{sin}\left(\Omega t_{\text{1}}+\theta \right)-\alpha_{r}u_{r}, \end{array}$$ (6)

where the dot denotes time differentiation with respect to the dimensionless time t_hs,_ which is defined as follows:


$$\begin{array}{c} {\text{t}}_{\text{hs}}=\omega_{hs}t,\;\text{and}\;\\ \omega_{hs}=\sqrt{\frac{k_{hs}}{m_{c}}}. \end{array}$$ (7)

Front and rear stiffness ratios β_tf_ and β_tr_ are introduced to describe the jumping nonlinearities in the front and rear tires. They depend on *Z*_uf_ and Z_ur_, which are defined as follows:



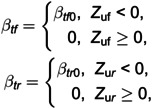

(8)


where β_tf0_ and β_tr0_ denote values of front and rear stiffness ratio when the tire maintains contact with the ground, that is, when Z_uf_ and Z_ur_ are negative. Otherwise, β_tf_ and β_tr_ are zero when front and rear tires lose contact with the ground. The delayed terms for the front and rear, u_f_ and u_r_, can be modelled as follows:


$$\begin{array}{c} u_{f}=\varepsilon_{f}\left(Z_{uf}(t)-Z_{uf}\left(t-\tau_{f}\right)\right)\;\text{and}\;\\ u_{r}=\varepsilon_{r}\left(Z_{ur}(t)-Z_{ur}\left(t-\tau_{r}\right)\right), \end{array}$$ (9)

where ε_f_, ε_r_, τ_f_, and τ_r_ denote front and rear feedback gains and delay time, respectively. Here, u_f_ and u_r_ are dimensionless control input forces for the front and rear suspension, respectively. Please see Appendix A.2 in S1 File for the detailed derivation. The motions of the front and rear unsprung masses, Z_uf_ and Z_ur_, respectively, are used for the delayed feedback.

Tables 1 and 2 summarize the parameters used in the simulations for the quarter-car and half-car models, respectively. The values of these parameters are based on typical agricultural off-road vehicles [26,29]. In this work, the solutions of the quarter- and half-car models are obtained numerically only. However, due to the presence of discontinuous nonlinearities and time delay, the analytical approach, e.g., the method of multiple scales [52–54], is difficult but may be attempted. MATLAB solver dde23 [55] is used for numerical integrations of the without- and with-delay systems with absolute tolerance of 10^−8^, and a maximum time step of 10^−3^_._ The dde23 solver uses an adaptive variable–time-step Runge–Kutta 2^nd^/3^rd^-order method with interpolation to solve delay differential equations. The initial state values for vehicle models are set to fixed points of the vehicle model without any road excitations, because vehicles typically start driving from a stationary state in practical driving scenarios. The initial state values are as follows: $Z_{s}(\text{0})=-\text{0}.\text{1}\text{1}\text{3}\text{0},\;Z_{u}(\text{0})=-\text{0}.\text{0}\text{1}\text{6}\text{1},\;{\dot{Z}}_{s}(\text{0})=\text{0},\;{\dot{Z}}_{u}=\text{0}$ and $Z_{sf}(\text{0})=-\text{0}.\text{0}\text{5}\text{6}\text{6},\;Z_{sr}(\text{0})=-\text{0}.\text{0}\text{5}\text{6}\text{6},\;\;Z_{uf}(\text{0})=-\text{0}.\text{0}\text{1}\text{0}\text{5},\;Z_{ur}(\text{0})=-\text{0}.\text{0}\text{1}\text{0}\text{5},{\dot{Z}}_{sf}(\text{0})=\text{0},\;{\dot{Z}}_{sr}=\text{0},\;{\dot{Z}}_{uf}(\text{0})=\text{0},\;{\dot{Z}}_{ur}=\text{0}$ for quarter- and half- car models respectively.

**Table 1:** Value of parameters for quarter-car model.

Parameters	Symbol	Value
Mass ratio	α	2.333
Stiffness ratio	β	10
Dimensionless gravity for quarter-car model	γ_q_	0.113
Damping ratio	ζ	0.155
Excitation amplitude	d_0_	0.025
Frequency ratio	Ω	7.5

**Table 2:** Value of parameters for half-car model.

Parameters	Symbol	Value
Front mass ratio	α_f_	2.333
Rear mass ratio	α_r_	2.333
Front suspension stiffness ratio	β_sf_	1
Rear suspension stiffness ratio	β_sr_	1
Front tire stiffness ratio	β_tf_	10
Rear tire stiffness ratio	β_tr_	10
Dimensionless gravity for half-car model	γ_h_	0.113
Front damping ratio	ζ_sf_	0.155
Rear damping ratio	ζ_sr_	0.155
Excitation amplitude	D_0_	0.025
Dimensionless inertia	ι	0.6
Dimensionless front length	λ_f_	0.5
Dimensionless rear length	λ_r_	0.5
Phase difference between front and rear	θ	−0.1
Frequency ratio	Ω	7.5

## 3 Results and discussion

### 3.1 Analysis of quarter-car model

#### 3.1.1 Model without delay.

**Fig 3:**
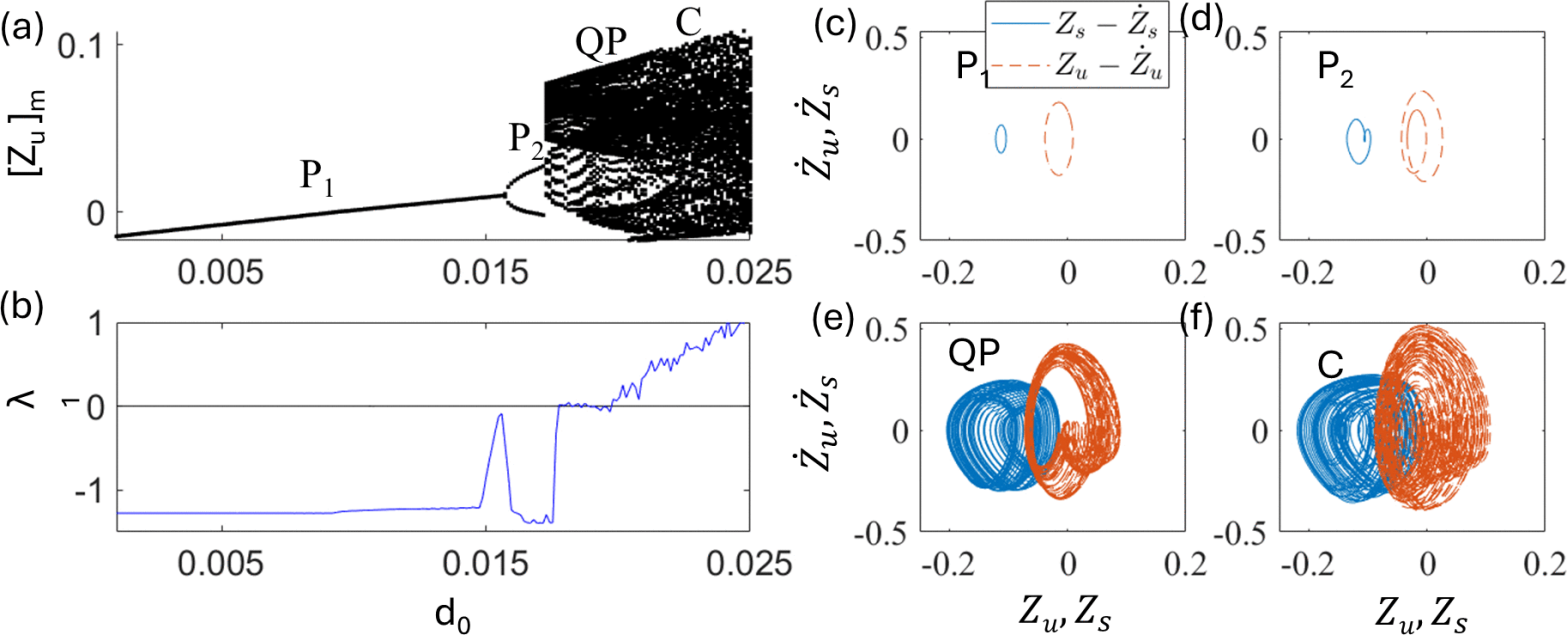
Plots of (a) bifurcation diagram and (b) largest nonzero Lyapunov exponents of the system without delay [Eq. (2)] as a function of excitation amplitude d_0_. The phase portraits of (c) period 1 (P_1_) at d_0 _= 0.015, (d) period 2 (P_2_) at d_0 _= 0.017, (e) QP at d_0 _= 0.02, and (f) chaos (C) at d_0 _= 0.025. The solid line (blue) is for $Z_{s}-{\dot{Z}}_{s}$ trajectory and the dashed line (red) is for $Z_{u}-{\dot{Z}}_{u}$.

In this section, a nonlinear quarter-car model without delay [u = 0, Eq. (2)] is investigated. A bifurcation diagram of the quarter-car model without delay is obtained by varying the excitation amplitude, d_0_. Fig 3a and 3b show the plot of the bifurcation diagram obtained by plotting extrema of Z_u_ and largest nonzero Lyapunov λ_1,_ respectively, where excitation amplitude d_0_ is varied.

As the excitation amplitude d_0_ increases, a period doubling bifurcation occurs at point d_0_ ~ 0.0155 from period 1 (P_1_) to period 2 (P_2_), as shown in Fig 3c and 3d, respectively. Upon further increasing d_0_, a discontinuous transition occurs at point d_0_ ~ 0.0175. This bifurcation is considered to be a crisis-type transition [56]. At this point, the vibration becomes a Quasi-periodic (QP) motion as shown in Fig 3e. Upon further increasing d_0_, the QP motion became chaotic (C), as shown in Fig 3f. Fig 3c, 3d, 3e, and 3f show the phase portraits of P_1_, P_2_, QP, and C for d_0_ = 0.015, 0.02,0.021, and 0.025, respectively. This bifurcation diagram indicates that the system exhibits various types of dynamics at different values of d_0_. This indicates that even a slight change in the road excitation amplitude d_0_ can trigger abrupt transitions in the system dynamics, leading to significantly larger vibration amplitudes. Therefore, road design and maintenance should be carefully managed to prevent such crisis-type transitions. Because of the presence of chaotic motion, controlling dynamics is important for improving the stability of vehicles.

**Fig 4:**
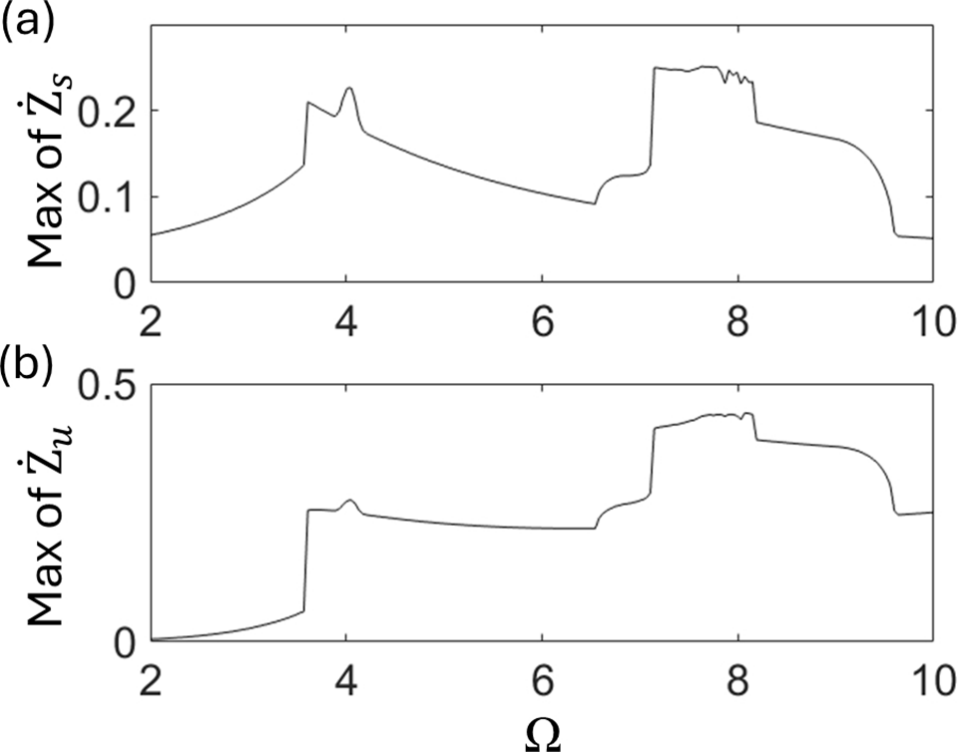
Plots of frequency response curves of (a) ${\dot{Z}}_{s}$ and (b) ${\dot{Z}}_{u}$.

To identify stability boundaries of the nonlinear quarter-car model, the frequency response curves of ${\dot{Z}}_{s}$ and ${\dot{Z}}_{u}$ are plotted in Fig 4a and 4b, respectively. Discontinuous increases in vibration amplitude are observed at Ω = 3.53 and 7.08, respectively. These indicate that some instabilities, such as bifurcations, appear at these points. In particular, the highest peak near Ω ~ 7.08 to 8.0 exhibits noticeable fluctuations, suggesting the presence of chaotic vibrations in this parameter range.

**Fig 5:**
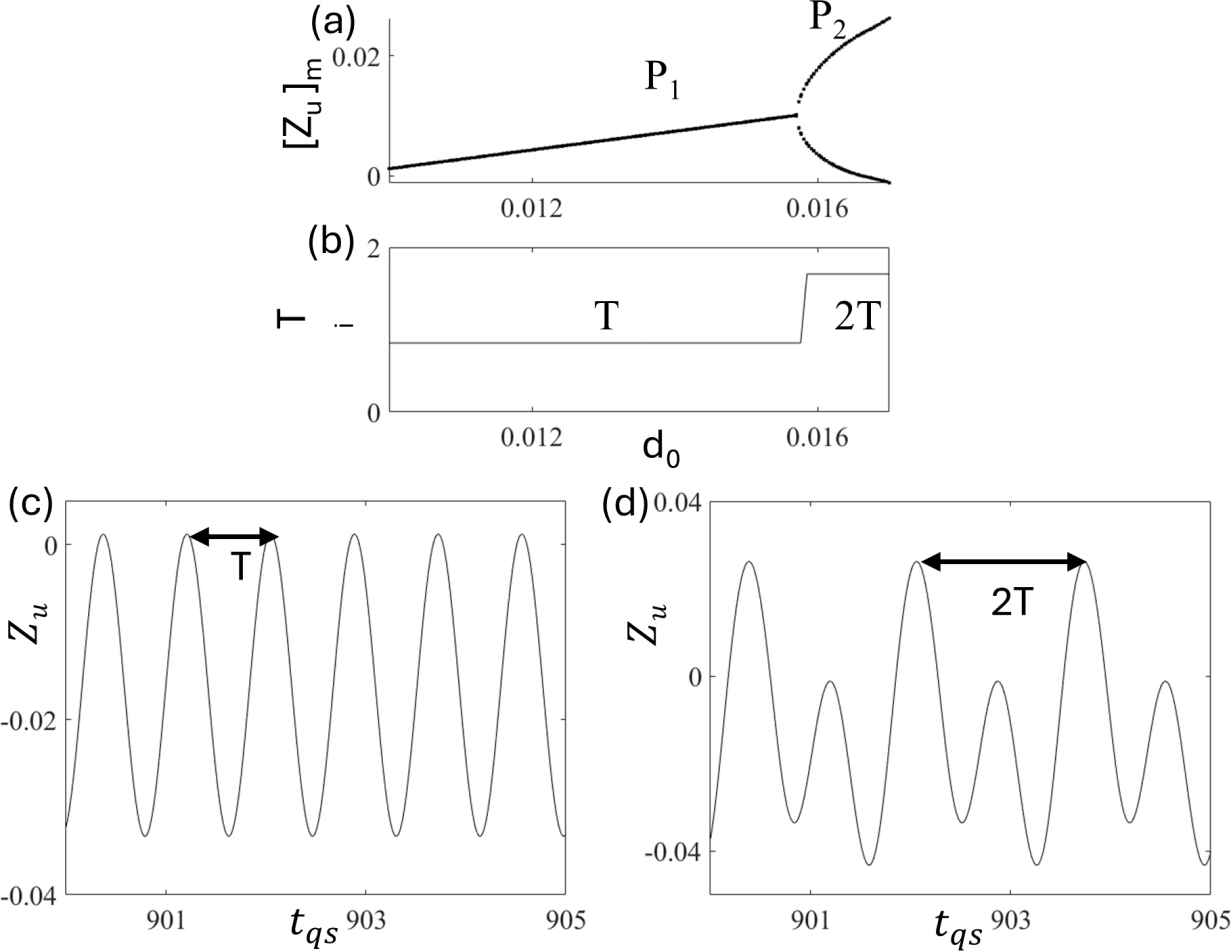
Plots of (a) the bifurcation diagram and (b) the time periods as a function of d_0_. The time series of periodic motions (c) P_1_ at d_0 _= 0.01 and (d) P_2_ at d_0 _= 0.017.

To characterize the periodic motions, Fig 5a and 5b show the bifurcation diagram and the corresponding interval of the period-i motion T_i,_ respectively, as a function of d_0_. The calculation of the time periods of period-i motion, T_i_, is indicated by the arrow in the time series in Fig 5c and 5d, where the time series of periodic motions, P_1_ and P_2,_ at d_0_ = 0.015 and 0.017, are shown, respectively. Here, we found that the interval of period-1 motion T_1_ equals the forcing period T = 2π/Ω = 0.838. However, after the period doubling bifurcation, the interval of period-2 motion T_2_ doubles the forcing period, that is, 2T = 1.676. These results indicate that the time periods of P_i_ motion T_i_ are equal to 2πi/Ω.

**Fig 6:**
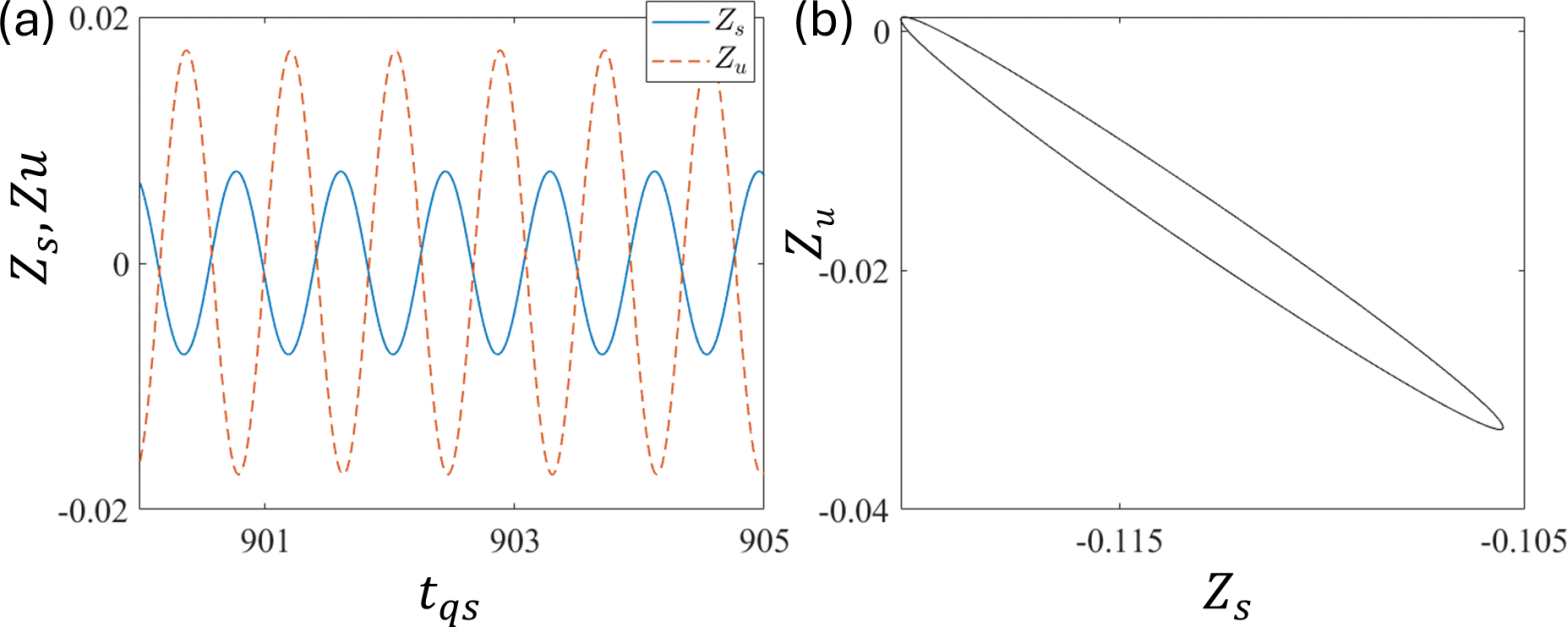
Plots of (a) the time series of Z_s_ (solid line) and Z_u_ (dashed line) and (b) the corresponding phase portrait at d_0 _= 0.01.

The quarter-car model has two degrees of freedom: sprung mass motion Z_s_ and unsprung mass motion Z_u_. Here, Z_s_ and Z_u_ are in vertical alignment, that is, the upper and lower distances, respectively. To understand the correlation between the dynamics of these alignments, Fig 6a and 6b show the time series of Z_s_ (solid line) and Z_u_ (dashed line) (subtracted from their means to see the correlation properly) with time and relative phase portraits in the Z_s_- Z_u_ plane, respectively, at d_0 _= 0.01. Fig 6a clearly shows that Z_s_ and Z_u_ exhibit out-of-phase motions, as confirmed in (b). Out-of-phase motion is physically natural for the vehicle vibration mode.

#### 3.1.2 Model with delay.

**Fig 7:**
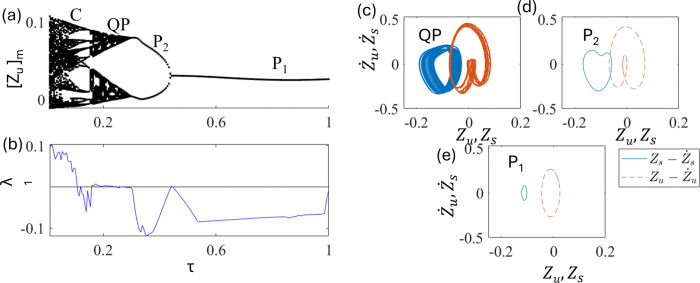
Plots of (a) bifurcation diagram and (b) largest nonzero Lyapunov exponent as a function of τ at the fixed d_0_ = 0.025 and ε = 1.0. The phase portraits of (c) QP at τ = 0.2, (d) period-2 P_2_ at τ = 0.35, and (e) period-1P_1_ at τ = 0.8. The solid line (blue) is for $Z_{s}-{\dot{Z}}_{s}$ trajectory and the dashed line (red) is for $Z_{u}-{\dot{Z}}_{u}$.

In this section, we consider the nonlinear quarter-car model with delay τ, where the control input is modelled as $u=\varepsilon \left(Z_{u}(t)-Z_{u}(t-\tau )\right)$. Fig 7a and 7b show the bifurcation diagram and the largest nonzero Lyapunov exponent as a function of delay τ at fixed d_0 =_ 0.025 and ε = 1.0.

For a zero delay (shown in Fig 3a), the motion is chaotic. As control is switched on and τ is increased, as shown in Fig 6a and 6b, chaos is stabilized into periodic motions. QP, P_2,_ and P_1_ appear at approximately τ ~ 0.165, 0.3, and 0.43, respectively. The phase portraits of these motions are shown in Fig 3c, 3d, and 3e respectively. These indicate that the proper selection of delays can stabilize the chaotic motion into regular motions. As can also be seen, the amplitude of the oscillations, that is, Z_u_, was reduced by approximately half without delay. Hence, it is useful to control jumping to achieve a better ride.

**Fig 8:**
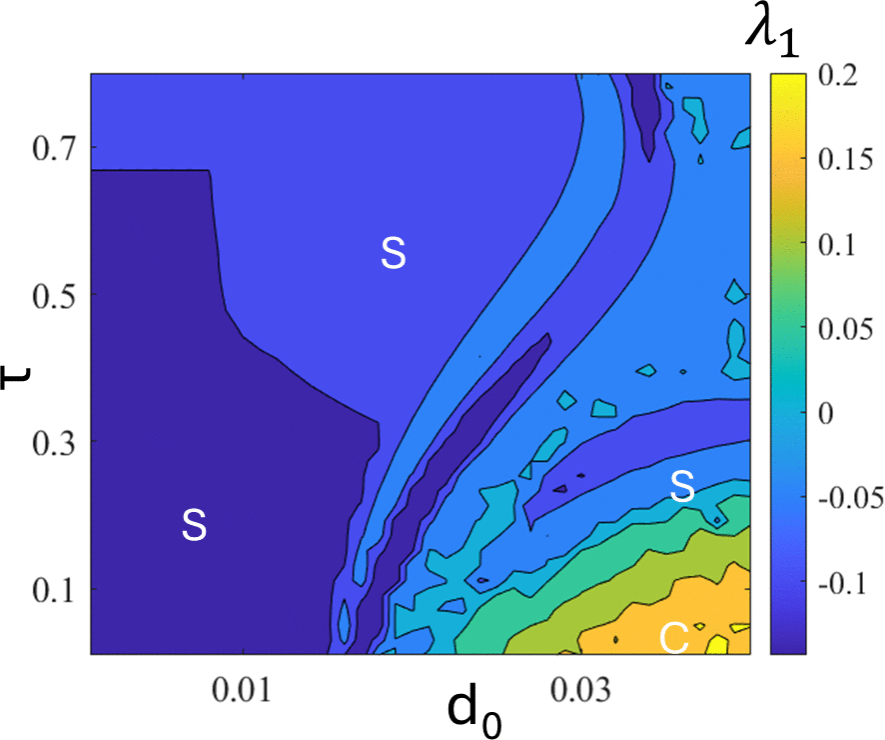
Heatmap of the largest nonzero Lyapunov exponent λ_1_ at ε = 1.0.

Fig 8 shows the heatmap of the largest nonzero Lyapunov exponent to observe the effect of parameters d_0_ and delay τ. Here, chaotic motions (C) are observed in lower τ and higher d_0,_ whereas periodic or stabilized motions (S) are observed at higher τ and lower d_0_. This indicates that the delay term successfully stabilizes chaos into regular motions for a wide range of parameters; hence, this technique can be used experimentally, where the control parameters fluctuate unavoidably. Time-delayed feedback is known to physically suppress vibrations [50,51]. In the numerical simulations conducted in this study, it effectively reduces vibration levels in the quarter-car model and stabilizes chaotic oscillations.

**Fig 9:**
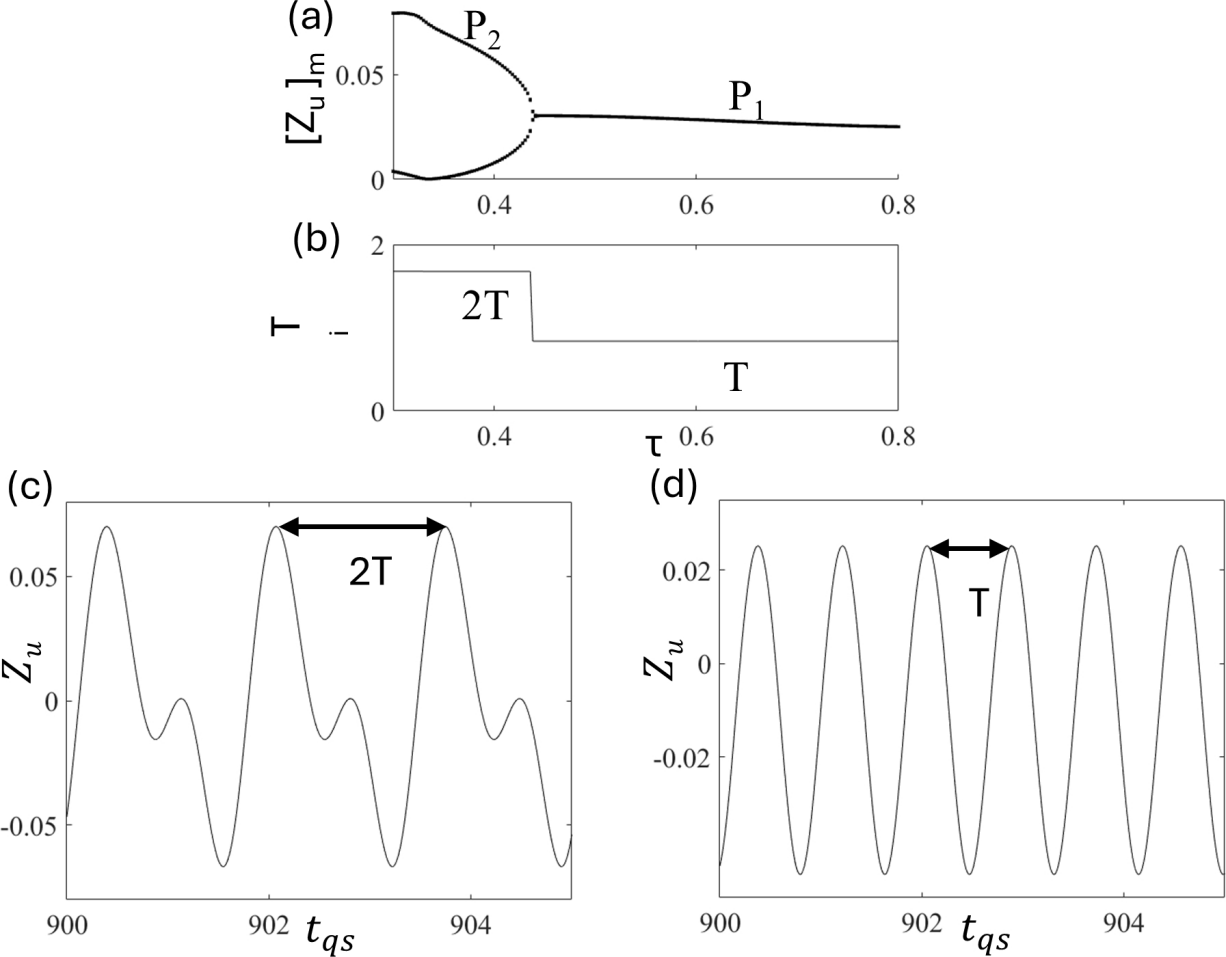
Plots of (a) bifurcation diagram and (b) time periods of period-i motion T_i_ as a function of τ at d_0_ = 0.025 and ε = 1.0. Time series of (c) period-2 P_2_ at τ = 0.35 and (d) period-1 P_1_ at τ = 0.8.

Fig 9a and 9b show the bifurcation diagram and time periods of period-i motion T_i_ as a function of τ to understand the period-i motion in the presence of excitation and delay. We observe that P_2_ (Fig 9c at τ = 0.35) becomes P_1_ (Fig 9d at τ = 0.8) via reverse period doubling bifurcation. Period-i remains constant. However, a jump occurred at the bifurcation point. Similarly, in Fig 5 (without delay), the intervals of peaks T_1_ and T_2_ were T = 0.838 and 2T = 1.676, respectively. These indicate that the relationship T_i_ = 2πi/Ω holds also in the quarter-car model with delay. Hence, the periodic relationship is completely dependent on the excitation period. Therefore, we conclude that although delay time τ affects stabilization of period-i motion, interval T_i_ majorly depends on forcing frequency Ω.

**Fig 10:**
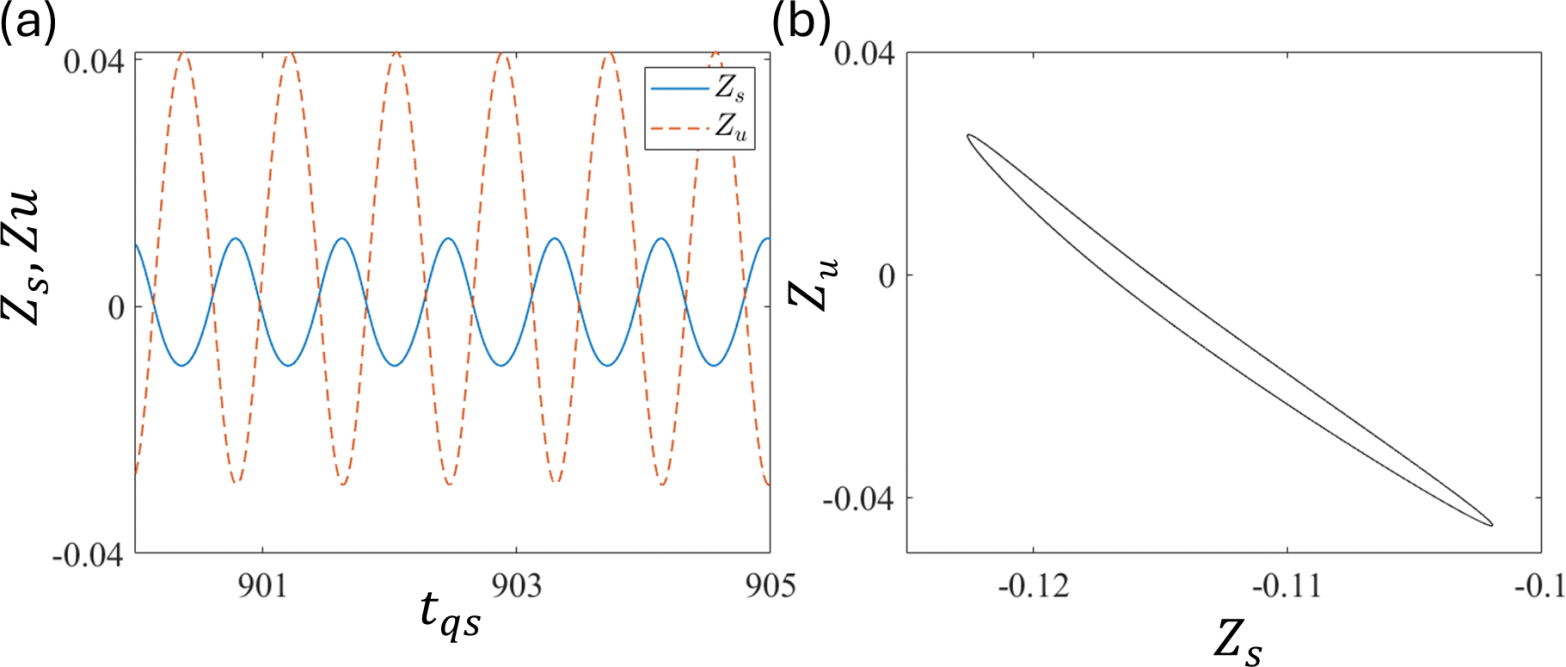
Plots of (a) time series Z_s_, and Z_u_ and (b) phase portrait at ε = 1.0 and τ = 0.8.

Fig 10a and 10b show the trajectory and phase portrait for period-1 to observe the phase correlation between Z_u_ and Z_s_. This shows that they are having out-of-phase motion at ε = 1.0 and τ = 0.8. This is similar to the case without delay. We also checked other parameter regions and found a similar out-of-phase relationship, indicating that out-of-phase motion is natural in the quarter-car model. Similar to the system without delay, in-phase motions were not observed in the quarter model with delay.

### 3.2 Analysis of half-car model

#### 3.2.1 Model without delay.

**Fig 11:**
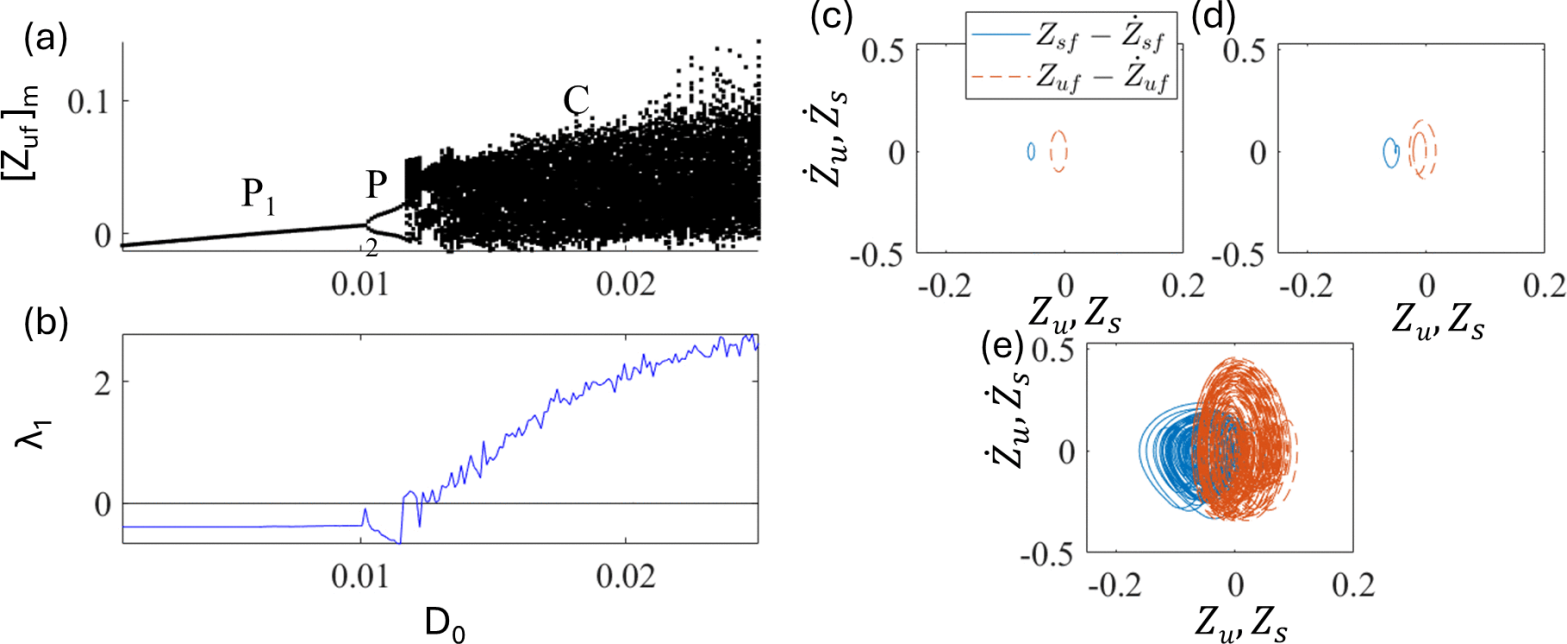
Plots of (a) bifurcation diagram and (b) largest nonzero Lyapunov exponent of the system without delay as a function of excitation amplitude d_0_. The phase portrait of (c) P_1_ at d_0_ = 0.008, (d) P_2_ at d_0_ = 0.011, and (e) C at d_0_ = 0.025. The solid line (blue) is for $Z_{sf}-{\dot{Z}}_{sf}$ trajectory and the dashed line (red) is for $Z_{uf}-{\dot{Z}}_{uf}$.

In this section, a half-car model without a delay [u_f_ = u_r_ = 0, Eq. (8)] is investigated. In this study, both front and rear excitation are considered almost the same, i.e., θ = −0.1. Fig 11a and 11b show the plots of the bifurcation diagram and the largest nonzero Lyapunov exponent as a function of d_0_.

As d_0_ is increased, a period doubling bifurcation occurs at point d_0_ ~ 0.011. With a further increase in d_0_, a discontinuous transition occurred at point d_0_ ~ 0.0135, and chaotic vibrations are observed. Fig 11c, 11d, and 11e show the phase portraits of P_1_, P_2_, and C in the half-car model at d_0_ = 0.008, 0.011, and 0.025, respectively. These indicate that the qualitative characteristics of the bifurcations in the nonlinear half-car model were similar to those in the quarter-car model, as shown in Fig 3a.

**Fig 12:**
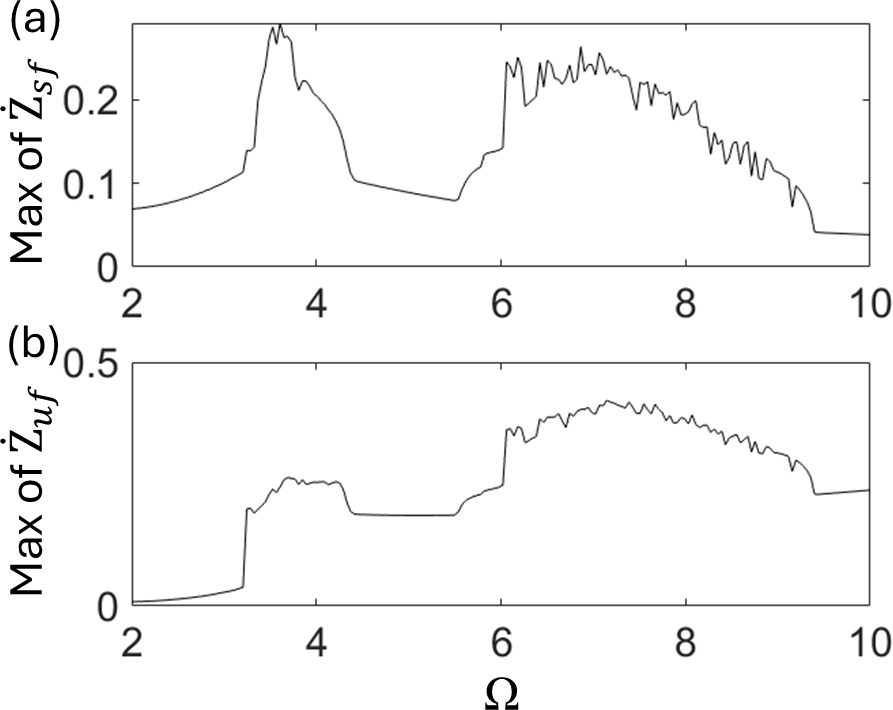
Plots of frequency response curves of (a)${\dot{Z}}_{sf}$ and (b)${\dot{Z}}_{uf}$.

To identify the stability boundary of the nonlinear half-car model, the frequency response curves of ${\dot{Z}}_{s}$and ${\dot{Z}}_{u}$are plotted in Fig 12a and 12b, respectively. Discontinuous increases in vibration amplitude are observed at Ω = 3.15 and 6.01, respectively. The qualitative characteristics of these frequency response curves are consistent with those shown in Fig 4, indicating that rich bifurcation transitions also occur in the nonlinear half-car model. The peak transitions from Ω = 7.08 to 4.1 from Ω = 6.01 to 9.2, both fluctuate, suggesting the presence of chaotic vibrations in these parameter regions. These results imply that chaotic oscillations are more likely to occur in the half-car model than in the quarter-car model.

**Fig 13:**
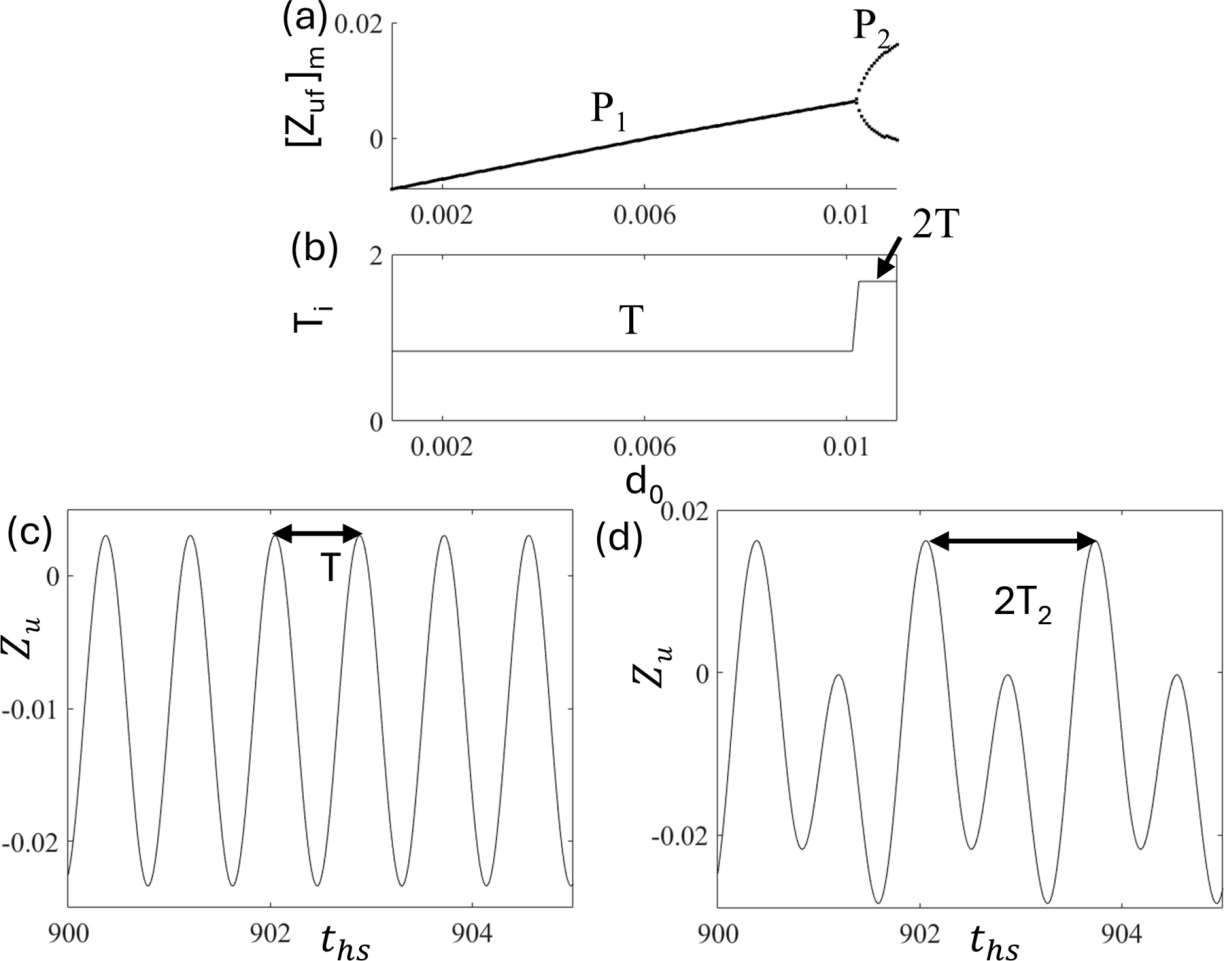
Plots of (a) bifurcation diagram and (b) time periods T_i_ as a function of d_0_, Time series of periodic motions (c) P1 at d_0_ = 0.008 and (d) P2 at d_0_ = 0.011.

To characterize the periodic motions, Fig 13a and 13b show the bifurcation diagram and time period of period-i motion T_i,_ respectively, which clearly show that the time period of period-i remains constant in P_1_ [Fig 13c] and P_2_ [Fig 13d]. However, a clear jump is observed, similar to that shown in Fig 5, for the quarter-car model. Similarly, in the half-car model, the time periods of the periodic motions T_1_ and T_2,_ are T = 0.838 and 2T = 1. 676. These indicate that the relationship T_i_ = 2πi/Ω holds in half-car model without delay.

**Fig 14:**
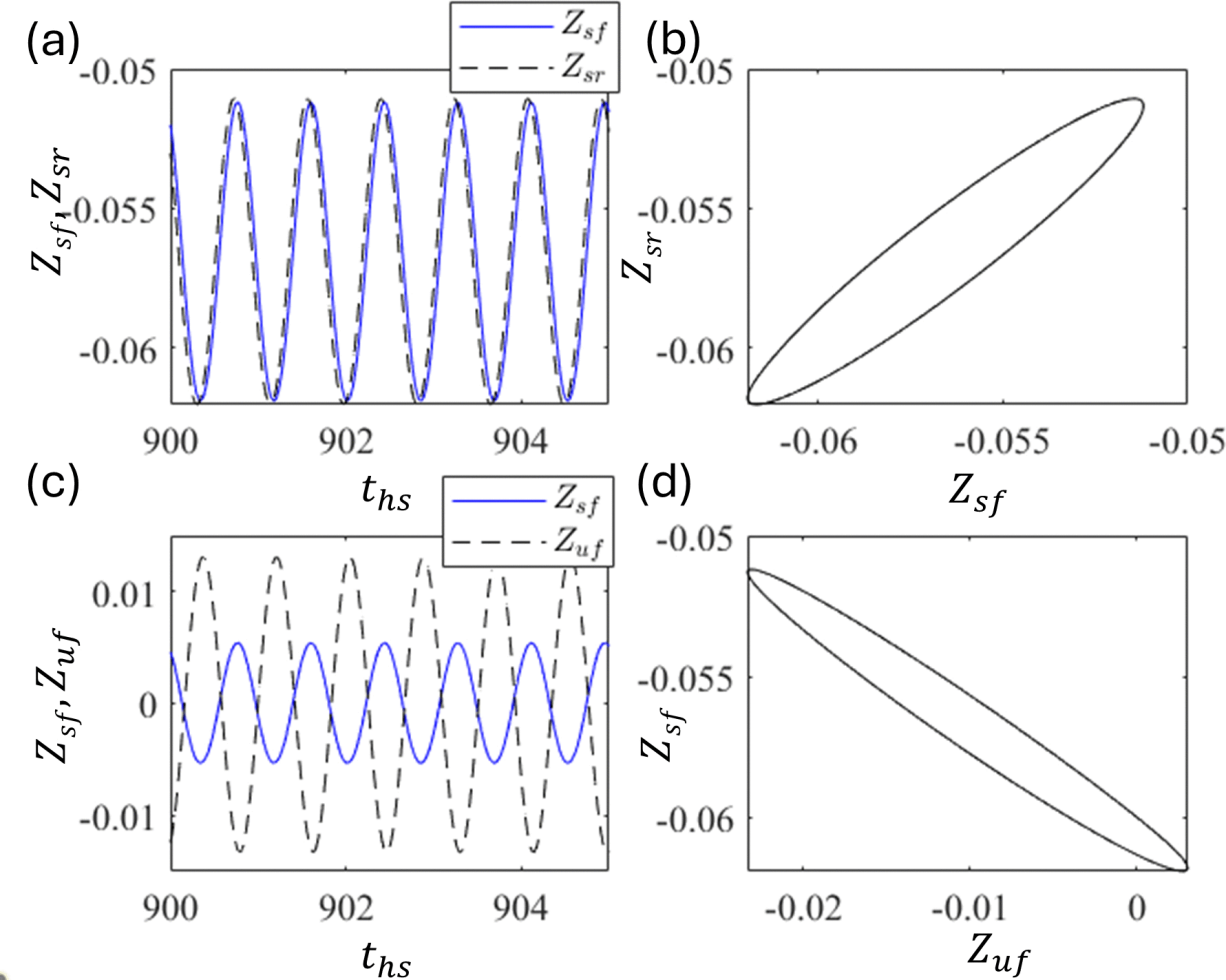
Plots of (a) time series of Z_sf_ and Z_sr_ and (b) corresponding phase portrait (b). (c) Time series of Z_sf_ and Z_uf_ and (d) corresponding phase portrait at d_0_ = 0.008.

As observed in the quarter-car model, the out-of-phase motion between Z_u_ and Z_s_, that is, vertical alignment, is natural. However, in the half-car model, apart from the vertical alignment, we have a horizontal alignment. Z_sf_ and Z_sr_ are in a horizontal alignment, that is, front and rear, whereas Z_sf_ and Z_uf_ are in a vertical alignment, that is, upper and lower, respectively. As shown in Fig 14, the time series (a) and (c) and phase portraits (b) and (d) at d_0_ = 0.008. It shows the in-phase motions between Z_sf_ and Z_sr_, along with out-of-phase motions between Z_sf_ and Z_sr_. These indicate that the horizontal alignment has an in-phase motion because the excitation functions d_f_ and d_r_ are almost the same, which is not desirable for practical purposes (however, this can be made out-of-phase with a delay, as discussed in the following sections).

#### 3.2.2 Model with delay.

**Fig 15:**
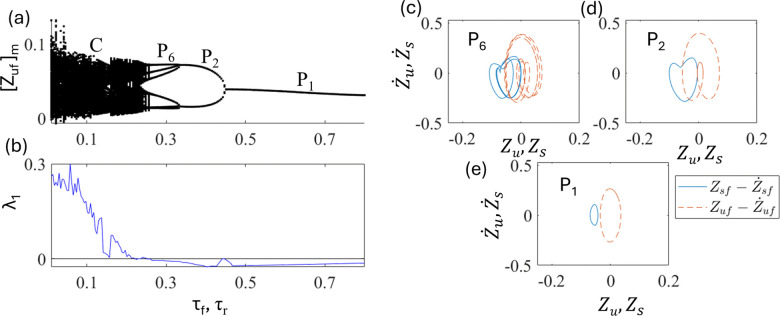
Plots of (a) bifurcation diagram and (b) largest nonzero Lyapunov exponent as a function of τ_f_ = τ_r_ = τ at d_0_ = 0.025 and ε = 1.5. The phase portraits of (c) Period-6 P_6_ at τ = 0.28, (d) Period-2 P_2_ at τ = 0.35, and (e) Period-1 P_1_ at τ = 0.6. The solid line (blue) is for $Z_{s}-{\dot{Z}}_{s}$ trajectory and the dashed line (red) is for $Z_{u}-{\dot{Z}}_{u}$.

In this section, a half-car model with a delay [Eq. (11)] is investigated. Fig 15a and 15b show the bifurcation diagram and largest nonzero Lyapunov exponent as a function of delays at fixed d_0 _= 0.025 and ε_f_ = ε_r _= ε = 1.5. As τ_f_ = τ_r _= τ increases, the chaos is stabilized into periodic motions. The periodic motions P_6_, P_2_, and P_1_ appear at τ ~ 0.25, 0.32, and 0.45, respectively. The qualitative characteristics of the bifurcations are similar to those of the quarter-car models. Fig 15c, 15d, and 15e show phase portraits of P_6_, P_2_, and P_1_ at τ = 0.28, 0.35, and 0.6, respectively. These indicate that an appropriate delay can stabilize chaotic motion, similar to the results of the quarter-car model. Besides amplitude of the motions becomes smaller as chaos stabilized into periodic motions.

**Fig 16:**
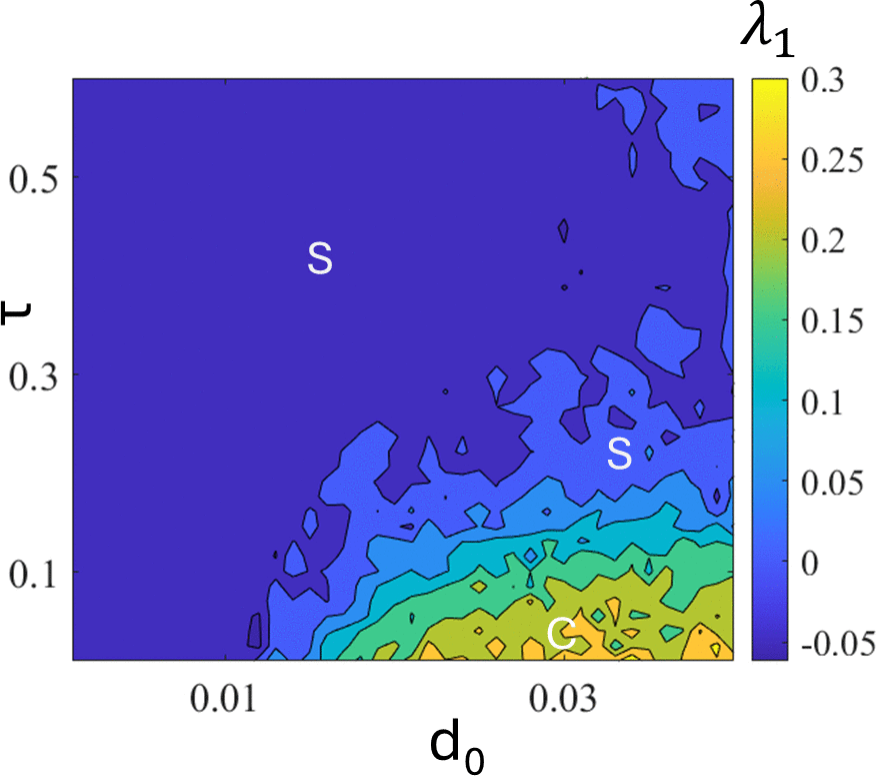
Heatmap of the largest nonzero Lyapunov exponentλ_1_ in the half-car model with delay.

Fig 16 shows a heatmap of the largest nonzero Lyapunov exponent to observe the effects of the parameters d_0_ and delay τ. Similarly, in the quarter model, chaotic motions (C) are observed in lower τ and higher d_0_, whereas periodic or stabilized motions (S) are observed in higher τ and lower d_0_. The results shown in Fig 16 are essentially equivalent to those in Fig 7, indicating that the mechanism by which time delay suppresses chaos is the same in both the quarter-car and half-car models.

**Fig 17:**
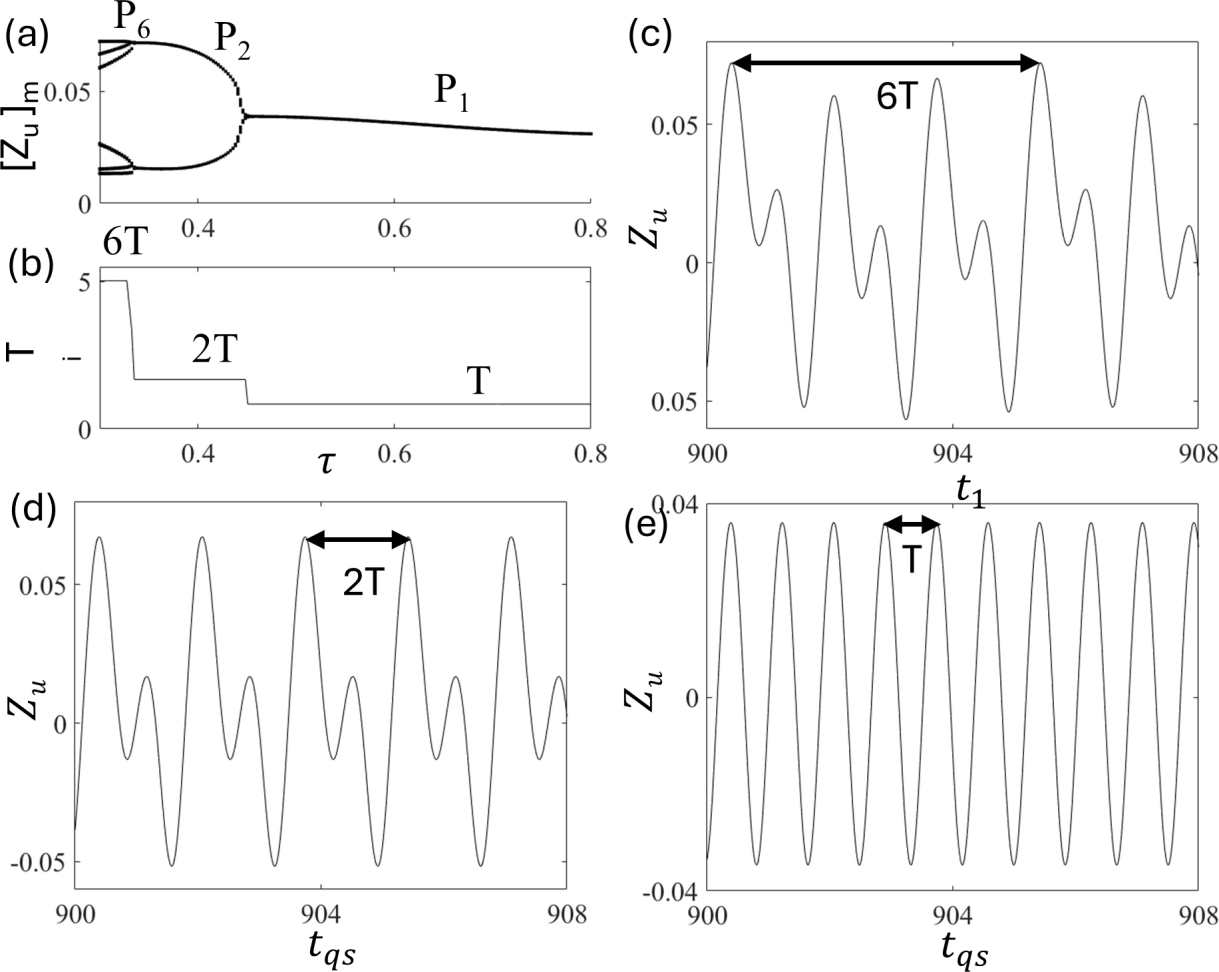
Plots of (a) bifurcation diagram and (b) time period of period-i motion T_i_ at d_0_ = 0.025 and ε = 1.5. The time series Z_uf_ of (c) P_6_ motion at τ = 0.3, (d) P_2_ motion at τ = 0.4, and (e) P_1_ motion at τ = 0.6.

Fig 17 and 17b show the plots of the bifurcation diagram and time period of the period-i motion T_i_. The time period T_6_, T_2_, and T_1_ are 6T = 5.265, 2T = 1.676, and T = 0.838, respectively. Fig 17c, 17d, and 17e show corresponding time series of P_1_, P_2,_ and P_6_ motion at τ = 0.3, 0.4, and 0.6, respectively. These indicate that the relationship T_i_ = 2πi/Ω holds in a half-car model with delay.

**Fig 18:**
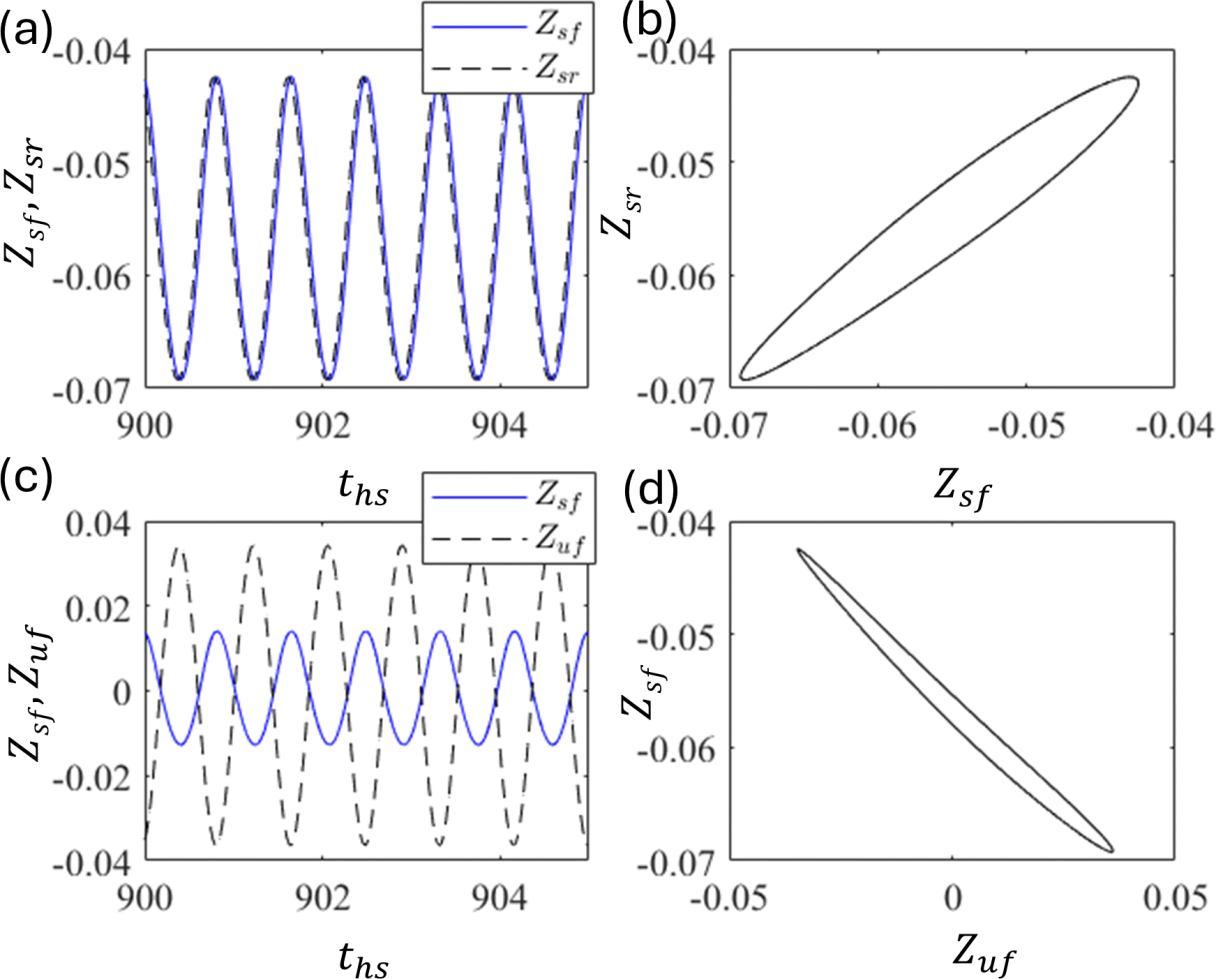
Plots of (a) time series of Z_sf_ and Z_sr_ and (b) corresponding phase portrait (b). (c) Time series of Z_sf_ and Z_uf,_ and (d) corresponding phase portrait at ε_f_ = ε_r_ = 1.5 and τ = 0.6.

Out-of-phase motion is observed in the horizontal alignment between Z_sf_ and Z_sr_ in the half-car model with a delay. As shown in Fig 18a and 18b, the in-phase motion is observed between Z_sf_ and Z_sr_, whereas Fig 16c and 16d show out-of-phase motion between Z_sf_ and Z_uf_ at ε = 1.5 and τ = 0.6. The motion between Z_sf_ and Z_sr_ is in-phase because the front and rear wheels are excited almost simultaneously. This result was similar to that of the quarter-car model.

**Fig 19:**
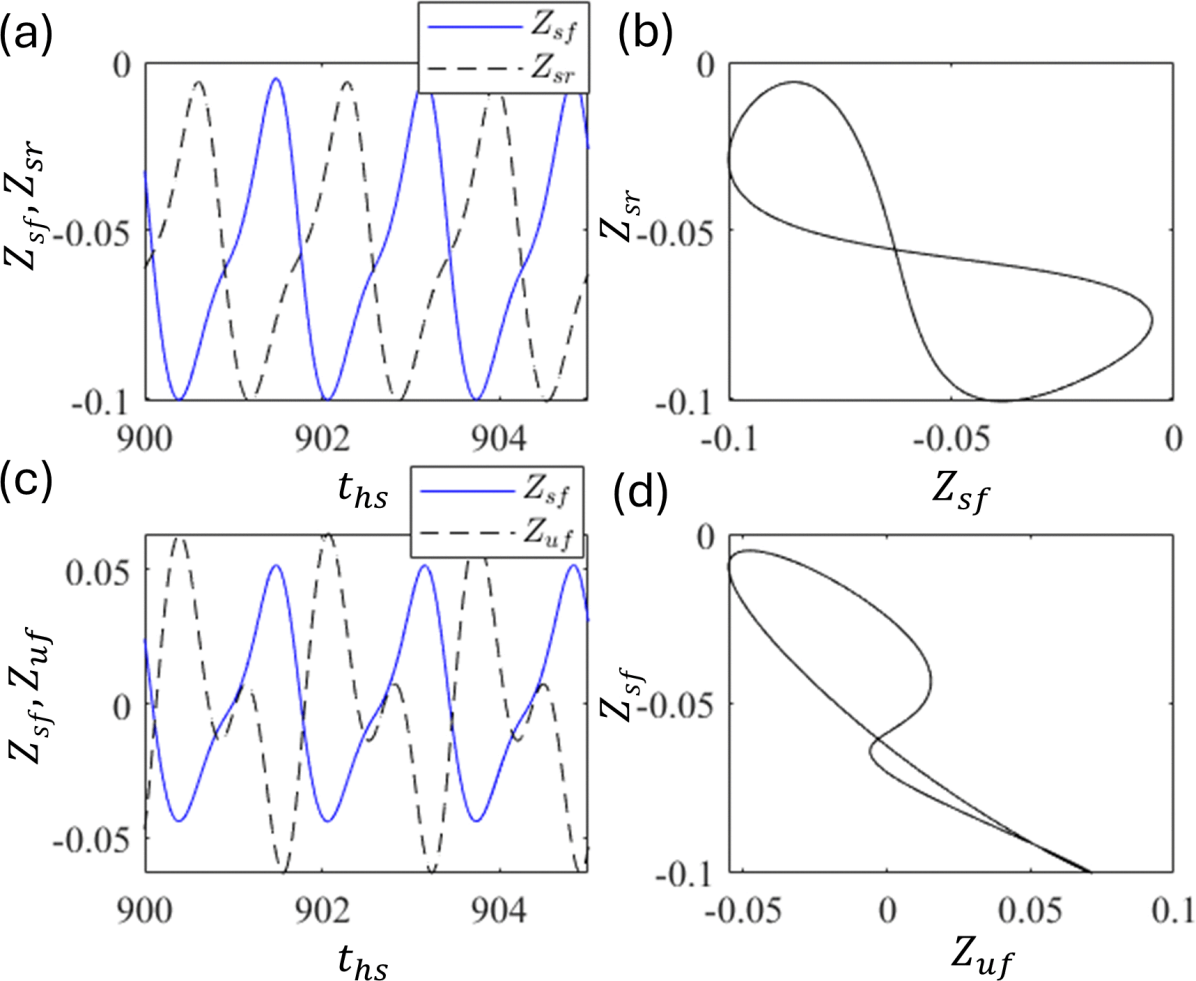
Plot of (a) time series of Z_sf_ and Z_sr_ and (b) corresponding phase portrait (b). (c) Time series of Z_sf_ and Z_uf_ and (d) corresponding phase portrait at ε = 1.5 and τ = 0.36.

The phase correlations shown in Fig 19a and 19b are the time series of Z_sf_ (solid line) and Z_sr_ (dashed line), and the corresponding phase portraits, respectively, at ε_f_ = ε_r_ = 1.5 and τ = 0.36. These indicate an out-of-phase relationship. Similarly, Fig 19c and 19d show the time series of Z_sf_ and Z_uf_ and the corresponding phase portraits, respectively. These are out-of-phase relationships.

Figs 18a, 18b and 19a, 19b suggest that at certain delays, horizontal alignment Z_sf_ and Z_sr_ show either in-phase or out-of-phase, depending on the delay τ. These suggest that the front and rear motions can be out-of-phase depending on the delay control parameters, even in the presence of simultaneous excitation between the front and rear. This transition is introduced by the time delay [57,58]. In practical applications, in-phase motion between the front and rear is undesirable because it can cause the front and rear tires to lose contact with the ground simultaneously, that is, free fall. This indicates strong instability in vehicle behavior, which can result in overturning accidents. Although out-of-phase motions (Fig 19) exhibit larger vibration amplitudes than in-phase motions (Fig 18), their opposing phases cause partial cancellation at the vehicle’s center of gravity, leading to a noticeable reduction in vibrations transmitted to the sprung mass. Therefore, delay τ should be tuned to induce out-of-phase motion between the front and rear in practical applications.

## 4. Conclusion

In this study, nonlinear quarter- and half-car models, both with and without time delays, were investigated. Sudden discontinuous transitions, interpreted as crisis-type transitions, were observed in both models, although the precise underlying mechanism remains unclear. A general relationship for period-i motions, T_i _= 2πi/Ω, was numerically identified in both vehicle models. This indicates that while the delay time τ influences the stabilization of period-i motions, the interval T_i_ is primarily governed by the forcing frequency Ω. This relationship was also confirmed in a forced Duffing oscillator, as shown in Appendix A.3 in S1 File, demonstrating the generality of the phenomenon. In-phase and out-of-phase motions induced by the delay were observed between the front and rear motions in the half-car model. We found that the front and rear motions can become out-of-phase for specific delay parameters, even under simultaneous excitations. From a practical standpoint, in-phase motion is undesirable because it can cause both the front and rear tires to lose contact with the ground simultaneously, leading to free-fall behavior and severe instability. Therefore, the delay τ should be tuned to promote out-of-phase motion to improve safety and controllability. The generality of these findings and their practical relevance should be further validated using more realistic vehicle models, such as a full-car model

This study identifies the stability regions of nonlinear quarter- and half-car models using bifurcation diagrams, Lyapunov exponents, and frequency response curves. In addition, time delay is shown to stabilize chaotic motions into periodic responses and to induce both in-phase and out-of-phase oscillations. Although these results highlight the importance of time delay in stabilizing the nonlinear dynamics of jumping vehicle models, further investigation is still needed. Future work should include experimental validation of the present findings, the application of analytical methods such as approximate solution techniques, and extension of the analysis to full-car models.

### Nomenclature and abbreviations

**Table:** 

Symbols	Meaning of symbols
*z*_*s*_	Vertical motion of sprung mass [m]
*z*_*u*_	Vertical motion of unsprung mass [m]
*Z*_*s*_	Suspension stroke [m]
*Z*_u_	Tire deflection [m]
*f*_s_	Suspension force [N]
*f*_t_	Tire force [N]
*f*_u_	Control force generated by active suspension [N]
m_s_	Sprung mass [kg]
m_u_	Unsprung mass [kg]
k_s_	Suspension stiffness [N m^-1^]
k_u_	Tire stiffness [N m^-1^]
c_s_	Suspension damping coefficient [N s m^-1^]
g	Gravitational acceleration [g]
d_0_	Sinus road function amplitude [m]
*t*	Time [s]
*t*_*qs*_	Scaled time for quarter-car model [rad]
*t*_*hs*_	Scaled time for half-car model [rad]
*t*_*0*_	Start time of fractional derivative [s]
ω_1_	Natural angular frequency of the sprung mass [rad s^-1^]
α	Mass ratio [-]
β	Stiffness ratio [-]
ζ	Damping ratio of the sprung mass [-]
γ_q_	Gravity term for quarter-car model [m]
Ω	Frequency ratio [-]
*z*_sf_	Vertical motion of front sprung mass [m]
*z*_*sr*_	Vertical motion of rear sprung mass [m]
*z*_*uf*_	Vertical motion of front unsprung mass [m]
*z*_*ur*_	Vertical motion of rear unsprung mass [m]
*Z*_*sf*_	Dimensionless front suspension stroke [-]
*Z*_*sr*_	Dimensionless rear suspension stroke [-]
*Z*_uf_	Dimensionless front tire deflection [-]
*Z*_uf_	Dimensionless rear tire deflection [-]
m_uf_	Front unsprung mass [kg]
m_ur_	Rear unsprung mass [kg]
k_tf_	Stiffness of front tires [kN m^ − 1^]
k_tr_	Stiffness of rear tires [kN m^ − 1^]
k_sf_	Front suspension stiffness [kN m^ − 1^]
k_sr_	Rear suspension stiffness [kN m^ − 1^]
c_tf_	Damping coefficient of front tires [N s m^ − 1^]
c_tr_	Damping coefficient of rear tires [N s m^ − 1^]
l_f_	Distance between the center of gravity of vehicle and front wheel [m]
l_r_	Distance between the center of gravity of vehicle and rear wheel [m]
α_f_	Front mass ratio [-]
α_r_	Rear mass ratio [-]
β_sf_	Front suspension stiffness ratio [-]
β_sr_	Rear suspension stiffness ratio [-]
β_tf_	Front tire stiffness ratio [-]
β_tr_	Rear tire stiffness ratio [-]
γ_h_	Dimensionless gravity for half model [-]
ζ_sf_	Front damping ratio [-]
ζ_sr_	Rear damping ratio [-]
D_0_	Excitation amplitude [-]
ι	Dimensionless inertia [-]
λ_f_	Dimensionless front length [-]
λ_r_	Dimensionless rear length [-]
θ	Phase difference between front and rear [-]
J	Pitch inertia [kg m^2^]
L	Wheelbase [m]
λ_1_	Largest nonzero Lyapunov exponent [-]
P_1_	Period 1
P_2_	Period 2
P_6_	Period-6
QP	Quasi-periodic
C	Chaotic

## Supporting information

S1 File Appendix. (PDF)

S2 File Program codes for nonlinear vehicle models. (PDF)
