# Supplementary material for: Effects of time delay on excited quarter- and half-car models with jumping nonlinearities
Source: PLoS One. 2026 Feb 5;21(2):e0340370. doi: 10.1371/journal.pone.0340370 (PMC12875590; doi:10.1371/journal.pone.0340370)
Supplement: S1 File — (PDF) [file pone.0340370.s001.pdf]

A.1 Derivation of the dimensionless quarter-car model

The derivation of the dimensionless quarter-car model is presented in detail in Appendix A.1. The equations of motion for the quarter-car model shown in Fig.1 are as follows:

$$m_s z_s'' = f_s + f_u - m_s g,$$
$$m_u z_u'' = f_t - f_s - f_u - m_u g, \qquad \text{(A.1)}$$

where the prime denotes differentiation with respect to time t, m_s, m_u, g, f_s, f_t, and f_u, denote the sprung mass, unsprung mass, gravitational acceleration, suspension force, tire force, and control forces generated by an active suspension, respectively. The suspension and tire forces are calculated as follows:

$$f_s = -k_s(z_s - z_u) - c_s(z_s' - z_u'),$$
$$f_t = -k_u(z_u - d), \qquad \text{(A.2)}$$

where k_s c_s, and k_u denote the suspension stiffness, suspension damping coefficient, and tire stiffness, respectively. Excitation function d is assumed to be a sine function as follows:

$$d(t) = d_0 \sin(\omega t), \qquad \text{(A.3)}$$

where d_0 and ω denote the amplitude and forcing frequency of the excitation function, respectively. To describe the jumping nonlinearity, the tire stiffness was introduced depending on the sign of the tire deflection z_2-d as follows:

$$k_t = \begin{cases} k_{t0}, & z_u - d < 0\text{: Tire keeps contact with road} \\ 0, & z_u - d \geq 0\text{: Tire loses contact with road} \end{cases}, \qquad \text{(A.4)}$$

where k_{t0} denotes the value of the tire stiffness. To derive the dimensionless form of the quarter-car model, the following dimensionless parameters were introduced:

$$Z_s = z_s - z_u,$$

$$Z_u = z_u - d,$$

$$t_{qs} = \omega_{qs} t,$$

$$\omega_{qs} = \sqrt{\frac{k_s}{m_s}},$$

$$u = \frac{f_u}{k_s},$$

$$\alpha = \frac{m_s}{m_u},$$

$$\beta = \frac{k_u}{k_s},$$

$$\zeta = \frac{c_s}{2\sqrt{m_s k_s}},$$

$$\gamma_q = \frac{g}{\omega_s^2} = \frac{m_s g}{k_s},$$

$$\Omega = \frac{\omega}{\omega_s} = \omega\sqrt{\frac{m_s}{k_s}}, \qquad \text{(A.5)}$$

To apply the above formulas, equations (A.1) become:

$$(Z_s'' + Z_u'') = -\frac{k_s}{m_s} Z_s - \frac{c_s}{m_s} {Z_s}' - g + d_0\omega^2 \sin\frac{\omega}{\omega_s} t_s + \frac{f_u}{k_s},$$

$$Z_u'' = -\frac{k_s}{m_u} Z_u + \frac{c_s}{m_u} Z_s' - \frac{k_u}{m_u} Z_u - g + d_0\omega^2 \sin\frac{\omega}{\omega_s} t_s - \frac{\alpha f_u}{k_s}. \qquad \text{(A.6)}$$

Using the relationship $Z''_s = \omega_s^2 \ddot{Z}_s$, dimensionless equations are as follows:

$$\ddot{Z}_s = -(1+\alpha)Z_s - 2\zeta(1+\alpha)\dot{Z}_s + \alpha\beta Z_u + (1+\alpha)u,$$
$$\ddot{Z}_u = \alpha Z_s + 2\zeta\alpha\dot{Z}_s - \alpha\beta Z_u - \gamma + d_0\Omega^2 \sin\Omega t_s - \alpha., \qquad \text{(A.7)}$$

Parameter switching (A.5) becomes:

$$\beta = \begin{cases} \beta, & Z_u < 0\text{: Tire keeps contact with road,} \\ 0, & Z_u \geq 0\text{: Tire loses contact with road.} \end{cases} \qquad \text{(A.8)}$$

A.2 Derivation of the dimensionless half-car model

In Appendix A.2, we present the derivation of a dimensionless half-car model. The equations of motion of the half-vehicle model shown in Fig.2 are as follows:

$$
\begin{aligned}
&m_c z'' = f_{sf} + f_{sr} + f_{uf} + f_{ur} - m_c g,\\
&J\phi'' = l_f(f_{sf} + f_{uf}) - l_r(f_{sr} + +f_{ur}),\\
&m_f z_f'' = f_{tf} - f_{sf} - f_{uf} - m_f g,\\
&m_r z_r'' = f_{tr} - f_{sr} - f_{ur} - m_r g, \qquad \text{(A.9)}
\end{aligned}
$$

where the prime denotes differentiation with respect to time *t*. The parameters m_c, m_f, m_r, J, l_f, and l_r, denote the sprung mass, front unsprung mass, rear unsprung mass, pitch inertia, distance between the front wheel and the center of gravity of the vehicle, and distance between the rear wheel and the center of gravity of the vehicle, respectively. Forces f_{sf}, f_{sr}, f_{tf}, f_{tf}, f_{uf}, and f_{ur} denote the front suspension, rear suspension, front tire, rear tire, front control, and rear control generated by the active suspension, respectively. The front and rear suspension and tire forces are as follows:

$$
\begin{aligned}
&f_{sf} = -k_{sf}(z + l_f\phi - z_f) - c_{sf}(\dot{z} + l_f\dot{\phi} - \dot{z}_f),\\
&f_{sr} = -k_{sr}(z - l_r\phi - z_r) - c_{sr}(\dot{z} + l_r\dot{\phi} - \dot{z}_r),\\
&f_{tf} = -k_{tf}(z_f - d_f),\\
&f_{tr} = -k_{tr}(z_r - d_r), \qquad \text{(A.10)}
\end{aligned}
$$

where k_{sf}, k_{sr}, c_{sf}, c_{sr} k_{tf}, and k_{tr} denote the front and rear suspension stiffnesses, front and rear suspension damping coefficients, and front and rear tire stiffnesses, respectively. To describe the jumping nonlinearity, the front and rear tire stiffnesses are switched depending on the sign of the front and rear tire deflections z_f-d_f and z_r-d_r as follows:

$$
k_{tf} = \begin{cases} -k_{tf0}, \text{if } (z_f - d_f) < 0\text{: Front tire keeps contact with road,} \\ \quad 0, \text{if } (z_f - d_f) \geq 0\text{: Front tire loses contact with road,} \end{cases}
$$

$$
k_{tr} = \begin{cases} -k_{tr0}, \text{if } (z_r - d_r) < 0\text{: Rear tire keeps contact with road,} \\ \quad 0, \text{ if } (z_r - d_r) \geq 0\text{: Rear tire loses contact with road,} \end{cases} \qquad \text{(A.11)}
$$

where k_{tf0} and k_{tr0} denote the front and rear tire stiffnesses, respectively. The front and rear excitation functions d_f and d_r are expressed as follows:

$$d_f = d_0 \sin(\omega t),$$
$$d_r = d_0 \sin(\omega t + \theta), \tag{A.12}$$

where θ denotes the phase difference between the front and the rear. Rear road excitation is delayed from front road excitation by θ. To derive the dimensionless form of the half-car model, dimensionless parameters are introduced as follows:

$$k_{hs} = \frac{k_{sf}+k_{sr}}{2},$$

$$\omega_{hs} = \sqrt{\frac{k_{hs}}{m_c}},$$

$$\mathrm{t_{hs}} = \omega_{hs} t,$$

$$\mathrm{L} = \mathrm{l_f} + \mathrm{l_r},$$

$$\alpha_f = \frac{m_c}{m_f},$$

$$\alpha_r = \frac{m_c}{m_r},$$

$$\iota = \frac{m_c L^2}{J},$$

$$\lambda_f = \frac{l_f}{L},$$

$$\lambda_r = \frac{l_r}{L},$$

$$\beta_{sf} = \frac{k_{sf}}{k_{hs}},$$

$$\beta_{sr} = \frac{k_{sr}}{k_{hs}},$$

$$\zeta_{sf} = \frac{c_{sf}}{2\sqrt{k_{hs} m_c}},$$

$$\zeta_{sr} = \frac{c_{sr}}{2\sqrt{k_{hs} m_c}},$$

$$\beta_{tf} = \frac{k_{tf}}{k_{hs}},$$

$$\beta_{tr} = \frac{k_{tr}}{k_{hs}},$$

$$u_f = \frac{f_{uf}}{k_{hs}L},$$

$$u_r = \frac{f_{ur}}{k_{hs}L},$$

$$\gamma_h = \frac{m_c g}{k_{hs}L},$$

$$Z_{sf} = \frac{z}{L} + \lambda_f \phi - \frac{z_f}{L},$$

$$Z_{sr} = \frac{z}{L} - \lambda_r \phi - \frac{z_r}{L},$$

$$Z_{uf} = \frac{z_f}{L} - \frac{d_f}{L},$$

$$Z_{ur} = \frac{z_r}{L} - \frac{d_{r,}}{L}$$

$$D_0 = \frac{d_0}{L}, \qquad \text{(A.13)}$$

Using the aforedefined parameters and variables, the following equations are derived:

$$\ddot{Z}_{sf} - \lambda_f \ddot{\phi} + \ddot{Z}_f = -\beta_{sf} Z_{sf} - 2\zeta_{sf} \dot{Z}_{sf} - \beta_{sr} Z_{sr} - 2\zeta_{sr} \dot{Z}_{sr} + u_f + u_r - \gamma_h,$$
$$\ddot{\phi} = -\beta_{sf} \lambda_f \iota Z_{sf} - 2\zeta_{sf} \lambda_f \iota \dot{Z}_{sf} + \beta_{sr} \lambda_r \iota Z_{sr} + 2\zeta_{sr} \lambda_r \iota \dot{Z}_{sr} + \lambda_f \iota u_f - \lambda_r \iota u_r,$$
$$\ddot{Z}_f = \ddot{Z}_{uf} + \ddot{D}_f = -\beta_{tf} \alpha_f Z_{uf} + \beta_{sf} \alpha_f Z_{sf} + 2\zeta_{sf} \alpha_f \dot{Z}_{sf} - \alpha_f u_f - \gamma_h,$$
$$\ddot{Z}_r = \ddot{Z}_{ur} + \ddot{D}_r = -\beta_{tr} \alpha_r Z_{ur} + \beta_{sr} \alpha_r Z_{sr} + 2\zeta_{sr} \alpha_r \dot{Z}_{sr} - \alpha_r u_r - \gamma_h, \qquad \text{(A.14)}$$

By solving the above simultaneous equations, the following dimensionless form of the half-car model is derived:

$$\ddot{Z}_{sf} = -\beta_{sf} A_f Z_{sf} - 2\zeta_{sf} A_f \dot{Z}_{sf} - \beta_{sr} A_{fr} Z_{sr} - 2\zeta_{sr} A_{fr} \dot{Z}_{sr} + \alpha_f \beta_{tf} Z_{uf} + A_f u_f + A_{fr} u_r,$$
$$\ddot{Z}_{ur} = -\beta_{sf} A_{fr} Z_{sf} - 2\zeta_{sf} A_{fr} \dot{Z}_{sf} - \beta_{sr} A_r Z_{sr} - 2\zeta_{sr} A_r \dot{Z}_{sr} + \alpha_r \beta_{tr} Z_{ur} + A_{fr} u_f + A_r u_r,$$
$$\ddot{Z}_{uf} = \alpha_f \beta_{sf} Z_{sf} + 2\alpha_f \zeta_{sf} \dot{Z}_{sf} - \alpha_f \beta_{tf} Z_{uf} - \gamma_h + D_0 \Omega^2 \ \sin(\Omega t_1) - \alpha_f u_f,$$
$$\ddot{Z}_{ur} = \alpha_r \beta_{sr} Z_{sr} + 2\alpha_r \zeta_{sr} Z_{sr} - \alpha_r \beta_{tr} Z_{ur} - \gamma_h + D_0 \Omega^2 \sin(\Omega t_1 + \theta) - \alpha_r u_r, \qquad \text{(A.15)}$$

where the following auxiliary parameters are introduced:

$$\mathrm{A}_f = \left(1 + \alpha_f + \lambda_f^2 \iota\right),$$
$$\mathrm{A}_{fr} = \left(1 - \lambda_f \lambda_r \iota\right),$$
$$\mathrm{A_f} = (1 + \alpha_r + \lambda_r^2 \iota). \qquad \text{(A.16)}$$

The front and rear stiffness ratios are as follows:

$$\beta_{tf} = \begin{cases} \beta_{tf0}, & Z_{\mathrm{uf}} < 0, \\ 0, & Z_{\mathrm{uf}} \geq 0, \end{cases}$$

$$\beta_{tr} = \begin{cases} \beta_{tr0}, & Z_{\mathrm{u}r} < 0, \\ 0, & Z_{\mathrm{u}r} \geq 0, \end{cases} \tag{A.17}$$

where β_{tf0} and β_{tr0} denote values of front and rear stiffness ratio when the tires maintain contact with the ground, that is, when Z_{uf} and Z_{ur} are negative.

A.3 Results in Duffing oscillator

The relationship between the interval of the peaks and the forcing frequency in the jumping vehicle model is also observed in the Duffing oscillator with and without delay. The following equations describe the Duffing system with the delay used in this section:

$$\ddot{x} + \gamma\dot{x} - x + x^3 = \mathrm{A}\cos\omega\mathrm{t} + u,$$
$$u = \varepsilon\big(x(t-\tau) - x(t)\big). \qquad \text{(A.18)}$$

In this study, γ is varied and ω = 2.9, and A = 2.8. First, the results for the Duffing oscillator without delay (u = 0) are presented. Fig. A.1 (a) and (b) show the plot of the bifurcation diagram and largest nonzero Lyapunov exponent when δ is varied. As γ is varied, chaotic, period-4, period-2, and period-1 motions are observed. Fig. A.1 (c), (d), (e), and (f) show the phase portraits of these motions, respectively.

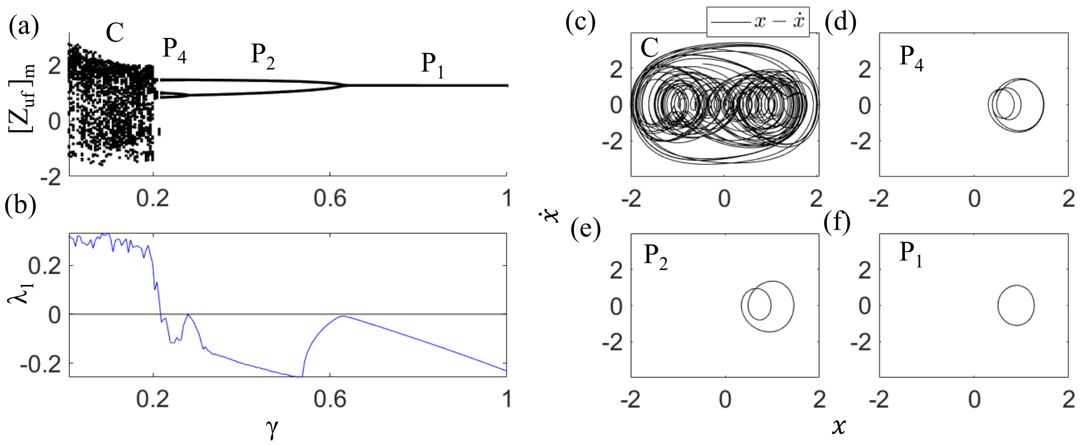

Fig. A.1 Plots of (a) Bifurcation diagram of the Duffing oscillator without delay varying excitation amplitude d_0, (b) largest nonzero Lyapunov exponents. Phase portrait of (c) C at γ = 0.1, (d) P_4 at γ = 0.23, (e) P_2 at γ = 0.4, and (f) P_1 at γ = 0.8.

Fig. A.2 shows the relationship between the interval of the peaks and period-i motions. As in Fig. A.2 (c), the interval of peaks for period-1 is $T_1 = T = 2\pi/\omega = 2.167$. These intervals double to $T_2 = 2T$ when they bifurcate from period-1 to period-2. Then, T_4 becomes 4T.

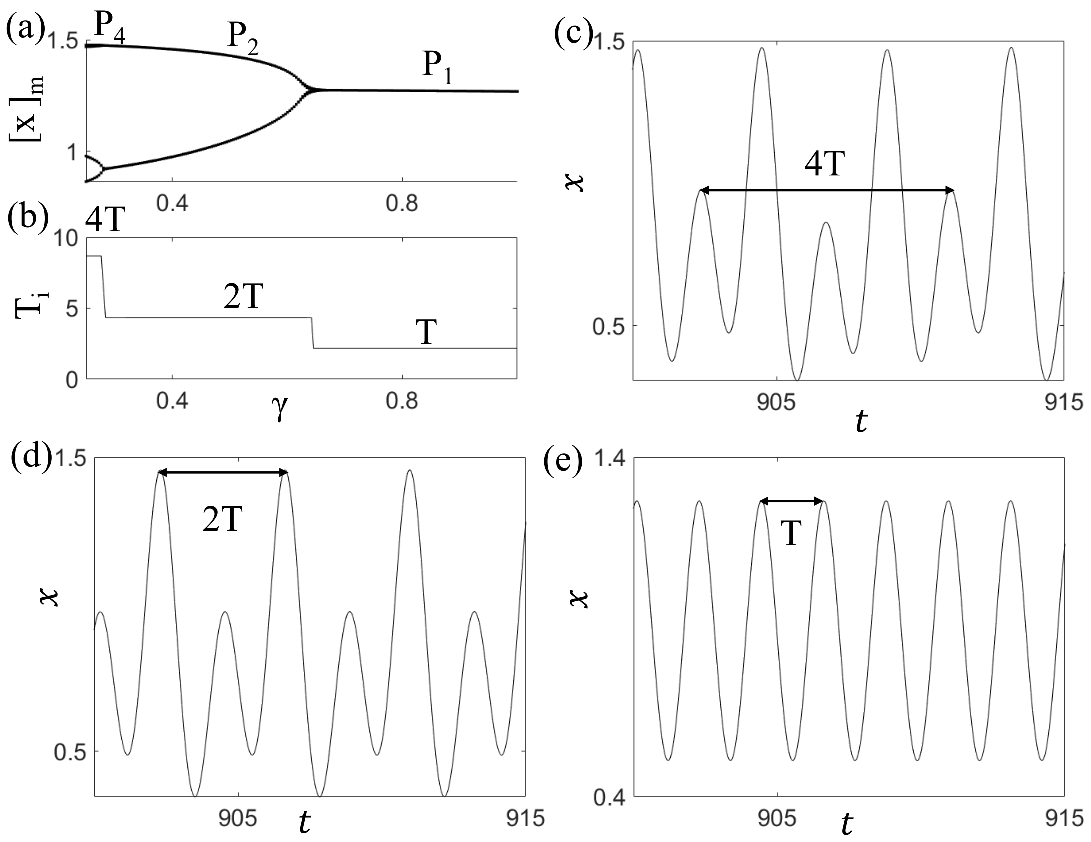

Fig. A.2 Plots of (a) bifurcation diagram and (b) interval between peaks. Time-series of (c) Period-4 at $\gamma = 0.23$, (d) Period-2 $\gamma = 0.4$, and (e) Period-1 $\gamma = 0.8$.

The results for the Duffing oscillator with a delay are then presented. Fig. A.3 (a) and (b) show the plot of the bifurcation diagram and largest nonzero Lyapunov exponent when τ is varied and ε and γ are fixed at 1.2 and 0.1, respectively. As τ is varied, chaotic, period-4, period-2, and period-1 motions are observed, similar to those in a system without delay. Fig. A.3 (c), (d), and (e) show the phase space of these motions, respectively.

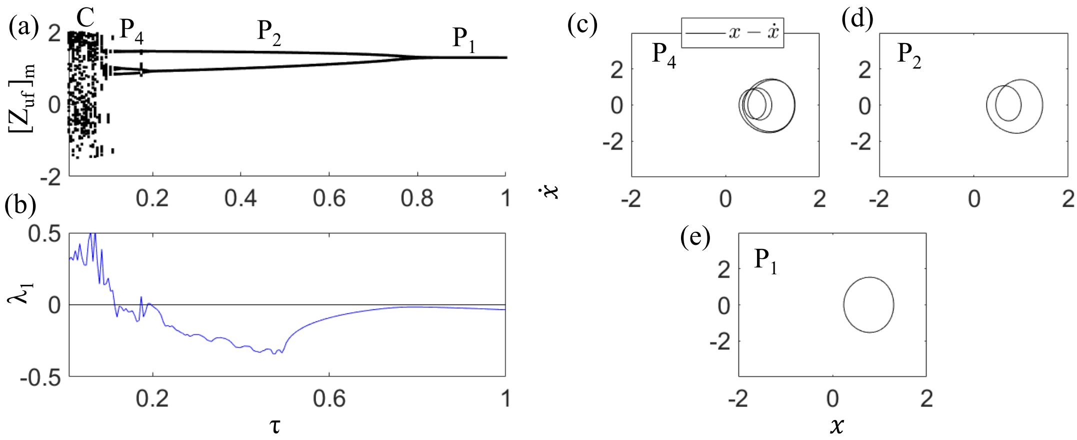

Fig. A.3 Plots of (a) bifurcation diagram of the Duffing oscillator with delay varying excitation amplitude d_0, (b) largest nonzero Lyapunov exponents. Phase portrait of (e) P_4 at $\tau = 0.15$, (d) P_2 at $\tau = 0.35$, and (c) P_1 at $\tau = 0.6$.

Fig. A.4 shows the relationship between the interval of peaks and period-i motions. As in Fig. A.4 (c), the interval of peaks for period-1 is $T_1 = T = 2\pi/\omega = 2.167$. These intervals become doubled to $T_2 = 2T$ when it bifurcates from period-1 to period-2. Then, T_4 becomes 4T.

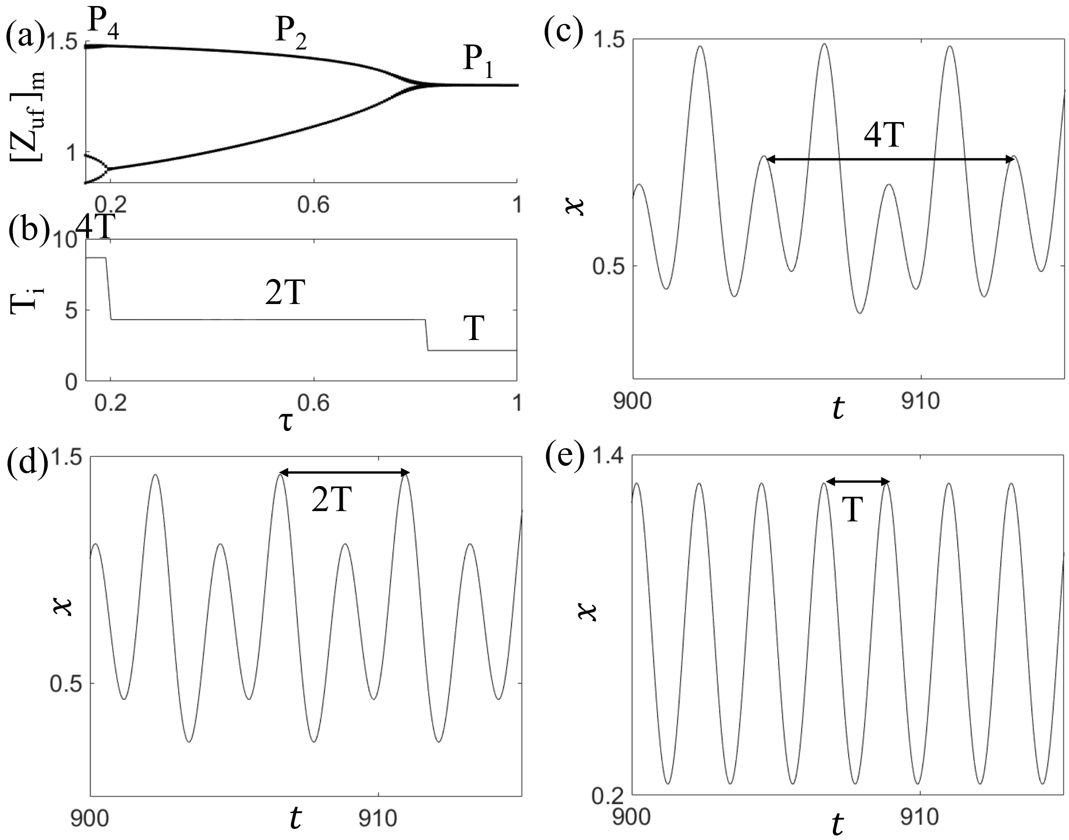

Fig. A.4 Plots of (a) bifurcation diagram at $\varepsilon = 1.2$ and (b) interval between peaks. Time-series of (c) Period-4 at $\tau = 0.15$, (d) Period-2 at $\tau = 0.35$, and (e) Period-1 at $\tau = 0.6$.

The above results demonstrate a general relationship, that is, $T_i = iT = 2\pi i/\omega$ holds regardless of the type of systems.

A.4 Noise robustness

In this section, the noise robustness of the obtained results is validated. The noise term is defined as follows:

$$
\begin{aligned}
&\eta \sim N\,(0,\,1),\\
&<\eta(t_1)>=0,\\
&<\eta(t_1) * \eta(t_1')>=\delta(t_1 - t_1'),
\end{aligned}
\qquad \text{(A.19)}
$$

The noise term is added in the quarter-car model as follows:

$$\ddot{Z}_u = \alpha Z_s + 2\zeta\alpha\dot{Z}_s - \alpha\beta Z_u - \gamma + d_0\Omega^2 \sin\Omega t_s + k_q\eta - \alpha, \qquad \text{(A.20)}$$

where k_q denotes the noise strength for the quarter-car model, which is set to 2.5% of the peak-to-peak value of Z_u when the chaotic vibration occurred at $d_0 = 0.025$ and without delay. The equations of motion with noise for the half-car model are as follows:

$$
\begin{aligned}
&\ddot{Z}_{uf} = \alpha_f\beta_{sf}Z_{sf} + 2\alpha_f\zeta_{sf}\dot{Z}_{sf} - \alpha_f\beta_{tf}Z_{uf} - \gamma_h + D_0\Omega^2\ \sin(\Omega t_1) + k_h\eta - \alpha_f u_f,\\
&\ddot{Z}_{ur} = \alpha_r\beta_{sr}Z_{sr} + 2\alpha_r\zeta_{sr}Z_{sr} - \alpha_r\beta_{tr}Z_{ur} - \gamma_h + D_0\Omega^2 \sin(\Omega t_1 + \theta) + k_h\eta - \alpha_r u_r,
\end{aligned}
\qquad \text{(A.21)}
$$

where k_h denotes the noise strength for the half-car model, which is set to 2.5% of the peak-to-peak value of Z_{uf} at which chaotic vibration occurs at $d_0 = 0.025$ without delay.

Figure A.5 (a) and (b) show the time series of periodic motions, P_1 and P_2, at $\tau = 0.8$ and 0.35, $d_0 = 0.025$ in the quarter-car model with delay, respectively. The results demonstrate that the relationship $T_i = iT = 2\pi i/\omega$ holds even in the presence of the noise. We also observe similar results in the half-car model. Therefore, the obtained relationship is robust and can be observed in a real system.

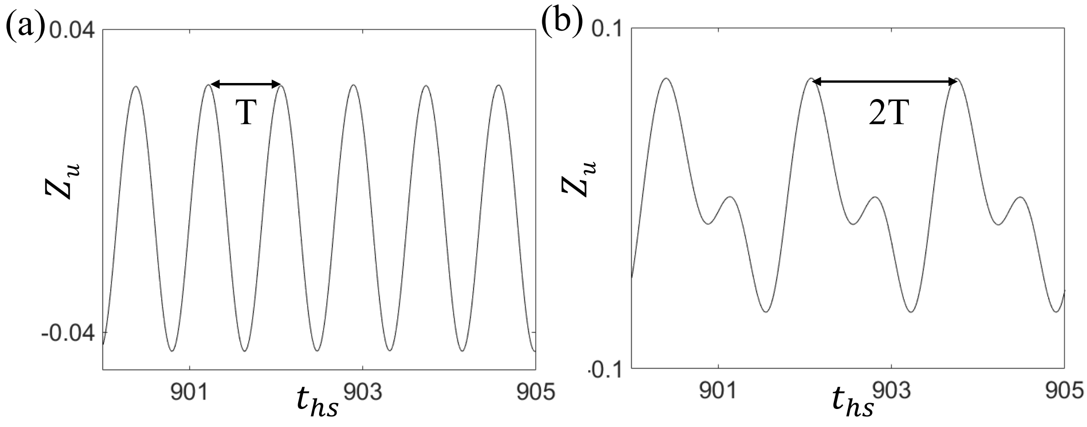

Fig. A.5 Timeseries of (a) Period-1 at τ = 0.8 and d_0 = 0.025, (b) Period-2 at τ = 0.35, and d_0 = 0.025

Figs.A.6 shows (a) and (b) show the out-of-phase motions between Z_{sf} and Z_{sr}, whereas Figs. A.6 (c) and (d) show out-of-phase motion between Z_{sf} and Z_{uf} at $\varepsilon_f = \varepsilon_r = 1.5$ and $\tau_f = \tau_r = 0.36$. The results demonstrate that the out-of-phase motions in the half-car model remained in the presence of noise.

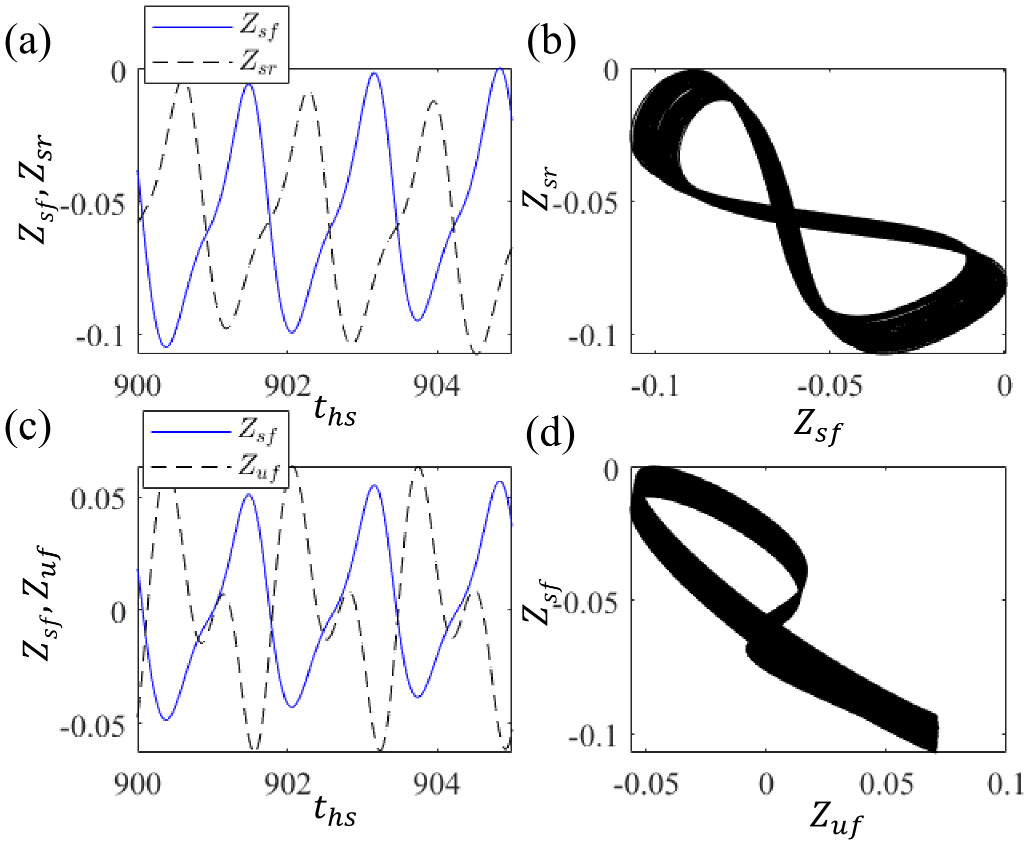

Fig. A.6 Plots of (a) time series of Z_{sf} and Z_{sr} and (b) corresponding phase portrait (b). (c) Time series of Z_{sf} and Z_{uf} and (d) corresponding phase portrait at $\varepsilon_f = \varepsilon_r = 1.5$ and $\tau_f = \tau_r = 0.36$.

The above numerical simulations demonstrate that the results obtained in the main manuscript have not changed and are thus robust to the presence of noise.
