# Supplementary material for: Effects of time delay on excited quarter- and half-car models with jumping nonlinearities
Source: PLoS One. 2026 Feb 5;21(2):e0340370. doi: 10.1371/journal.pone.0340370 (PMC12875590; doi:10.1371/journal.pone.0340370)
Supplement: S2 File — (PDF) [file pone.0340370.s002.pdf]

MATLAB program code for nonlinear quarter-car model

```
%% MATLAB initialization
clear all;close all;clc;
%% dimensionless params

alpha=2.333;
beta0=10;
beta=beta0;
zeta=0.1552;
gamma_g=0.113;
d0=0.025;

Omega=7;
Omega2=Omega^2;

T=100;
%% control params
eps = 0;
tau=0.8;
%% white gaussian noise
sigma_noise=0.0;
std_noise=sqrt(sigma_noise);

%% sim
opts=ddeset('AbsTol', 1e-8,MaxStep=1e-3);
tspan = [0 T];
x0 = [-0.113 -0.0161 0 0]';
sol = dde23(@(t,x,xd) derivs(t,x,xd,alpha,beta,zeta,gamma_g,d0,Omega,eps), ...
              tau,x0,tspan,opts);

%% res
z1=sol.y(1,:);
z2=sol.y(2,:);
z1_=sol.y(3,:);
z2_=sol.y(4,:);
```

```
t=sol.x;
%% functions
function dxdt = derivs(t,x,xd,alpha,beta,zeta,gamma,d0,Omega,eps)
    beta=(1-sign(x(2)))*beta/2;
    u=eps*(x(2)-xd(2));

    sigma_noise=0;
    std_noise=sigma_noise*0.025;
    eta=1*std_noise*randn(1,1);

    dxdt(1) = x(3);
    dxdt(2) = x(4);
    dxdt(3) = -(1+alpha)*x(1)+beta*alpha*x(2)-2*zeta*(1+alpha)*x(3)+(1+alpha)*u;
    dxdt(4) = alpha*x(1)-beta*alpha*x(2)+2*zeta*alpha*x(3)-
gamma+d0*Omega^2*sin(Omega*t)+eta-alpha*u;
    dxdt=dxdt';
end
```

MATLAB program code for nonlinear half-car model

```
%% Matlab Initialization
clc;clear all;close all;
tic;
%% Parameter for tractor with axial suspension
alpha_f=2.333;
alpha_r=2.333;
L=1;
iota=0.6;
lambda_f=0.5;
lambda_r=0.5;
beta_sf=1;
beta_sr=1;
zeta_sf=0.1552;
zeta_sr=0.1552;
beta_tf=10;
beta_tr=10;
gamma_h=0.1133;
theta=-1.9*pi*1;
Omega=7;
d0=0.025*1;

x0 = zeros(1,8);
x0(1)=-0.0566;
x0(2)=-0.0566;
x0(3)=-0.0105;
x0(4)=-0.0105;

eps_f=0*1.5;
eps_r=0*1.5;
tau_f=0.6;
tau_r=0.6;

%% sim params
T = 100;
```

```
opts=ddeset('AbsTol', 1e-8,MaxStep=1e-3);
tspan = [0 T];

sol = dde23(@(t,x,xd) derivs(t,x,xd, alpha_f, alpha_r,Omega, L, iota,lambda_f, lambda_r,...
                      beta_sf,beta_sr,zeta_sf,zeta_sr,beta_tf,beta_tr,...
                      gamma_h,d0,theta,eps_f,eps_r),...
                        [tau_f tau_r tau_f tau_r tau_f tau_r tau_f tau_r],x0,tspan,opts);

zsf=sol.y(1,:);
zsr=sol.y(2,:);
zuf=sol.y(3,:);
zur=sol.y(4,:);
zsf_=sol.y(5,:);
zsr_=sol.y(6,:);
zuf_=sol.y(7,:);
zur_=sol.y(8,:);
t=sol.x;
%% functions
function dxdt= derivs(t,x,xd, alpha_f, alpha_r, Omega, L, iota, lambda_f, lambda_r, ...
                                    beta_sf,beta_sr,zeta_sf,zeta_sr,beta_tf,beta_tr,...
                                    gamma_h,d0,theta,eps_f,eps_r)

    zsf   = x(1);
    zsr   = x(2);
    zuf   = x(3);
    zur   = x(4);
    zsf_ = x(5);
    zsr_ = x(6);
    zuf_ = x(7);
    zur_ = x(8);

    zsfd=xd(1);
    zsrd=xd(2);
    zufd=xd(3);
    zurd=xd(4);
    zsfd_=xd(5);
```

```
zsrd_=xd(6);
zufd_=xd(7);
zurd_=xd(8);

beta_tf=(1-sign(zuf))*beta_tf/2;
beta_tr=(1-sign(zur))*beta_tr/2;

Af=(1+alpha_f+lambda_f^2*iota);
Afr=(1-lambda_f*lambda_r*iota);
Ar=(1+alpha_r+lambda_r^2*iota);

uf=eps_f*(zuf-zufd);
ur=eps_r*(zur-zurd);

% noise
sigma_noise=0;
std_noise=0*0.2318*0.025;sqrt(sigma_noise);
eta_f=1*std_noise*randn(1,1);
eta_r=1*std_noise*randn(1,1);

dxdt(1) = zsf_;
dxdt(2) = zsr_;
dxdt(3) = zuf_;
dxdt(4) = zur_;
dxdt(5) = -beta_sf*Af*zsf-2*zeta_sf*Af*zsf_-beta_sr*Afr*zsr-2*zeta_sr*Afr*zsr_ ...
          +alpha_f*beta_tf*zuf+Af*uf+Afr*ur;

dxdt(6) = -beta_sf*Afr*zsf-2*zeta_sf*Afr*zsf_-beta_sr*Ar*zsr-2*zeta_sr*Ar*zsr_ ...
          +alpha_r*beta_tr*zur+Afr*uf+Ar*ur;

dxdt(7) = alpha_f*beta_sf*zsf+2*alpha_f*zeta_sf*zsf_-alpha_f*beta_tf*zuf ...
          -alpha_f*uf-gamma_h+d0*Omega^2/L*sin(Omega*t)+eta_f;
dxdt(8) = alpha_r*beta_sr*zsr+2*alpha_r*zeta_sr*zsr_-alpha_r*beta_tr*zur ...
          -alpha_r*ur-gamma_h+d0*Omega^2/L*sin(Omega*t+theta)+eta_r;
```

```
    dxdt=dxdt';
end
```
